# Discovery of prosimian and afrotherian foamy viruses and potential cross species transmissions amidst stable and ancient mammalian co-evolution

**DOI:** 10.1186/1742-4690-11-61

**Published:** 2014-08-04

**Authors:** Aris Katzourakis, Pakorn Aiewsakun, Hongwei Jia, Nathan D Wolfe, Matthew LeBreton, Anne D Yoder, William M Switzer

**Affiliations:** Department of Zoology, University of Oxford, Oxford, South Parks Road, Oxford, OX1 3PS UK; Laboratory Branch, Division of HIV/AIDS, National Center for HIV, Hepatitis, STD, and TB Prevention, Centers for Disease Control and Prevention, Atlanta, GA 30333 USA; Metabiota, One Sutter, Suite 600, San Francisco, CA 94104 USA; Program in Human Biology, Stanford University, Stanford, CA 94305 USA; Global Viral, One Sutter, Suite 600, San Francisco, CA 94104 USA; Mosaic (Environment, Health, Data, Technology), Yaoundé, Cameroon; Department of Biology, Duke University, Durham, NC 27708 USA

**Keywords:** Paleovirology, Endogenous retrovirus, Foamy virus, Co-evolution, PSFVgal, PSFVaye, ChrEFV

## Abstract

**Background:**

Foamy viruses (FVs) are a unique subfamily of retroviruses that are widely distributed in mammals. Owing to the availability of sequences from diverse mammals coupled with their pattern of codivergence with their hosts, FVs have one of the best-understood viral evolutionary histories ever documented, estimated to have an ancient origin. Nonetheless, our knowledge of some parts of FV evolution, notably that of prosimian and afrotherian FVs, is far from complete due to the lack of sequence data.

**Results:**

Here, we report the complete genome of the first extant prosimian FV (PSFV) isolated from a lorisiforme galago (PSFVgal), and a novel partial endogenous viral element with high sequence similarity to FVs, present in the afrotherian Cape golden mole genome (ChrEFV). We also further characterize a previously discovered endogenous PSFV present in the aye-aye genome (PSFVaye). Using phylogenetic methods and available FV sequence data, we show a deep divergence and stable co-evolution of FVs in eutherian mammals over 100 million years. Nonetheless, we found that the evolutionary histories of bat, aye-aye, and New World monkey FVs conflict with the evolutionary histories of their hosts. By combining sequence analysis and biogeographical knowledge, we propose explanations for these mismatches in FV-host evolutionary history.

**Conclusion:**

Our discovery of ChrEFV has expanded the FV host range to cover the whole eutherian clade, and our evolutionary analyses suggest a stable mammalian FV-host co-speciation pattern which extends as deep as the exafroplacentalian basal diversification. Nonetheless, two possible cases of host switching were observed. One was among New World monkey FVs, and the other involves PSFVaye and a bat FV which may involve cross-species transmission at the level of mammalian orders. Our results highlight the value of integrating multiple sources of information to elucidate the evolutionary history of viruses, including continental and geographical histories, ancestral host locations, in addition to the natural history of host and virus.

**Electronic supplementary material:**

The online version of this article (doi:10.1186/1742-4690-11-61) contains supplementary material, which is available to authorized users.

## Background

Foamy viruses (FVs) are complex retroviruses in the *Spumaretrovirinae* subfamily [1]. All known contemporary FVs cause persistent but non-virulent, asymptomatic infections exclusively among boreoeutherian mammals (Additional file 1: Table S1) [1]. The first FV was discovered in a macaque in 1954 [2] and was isolated in 1955 [3]. Shortly thereafter, numerous FVs were isolated from other boreoeutherian mammals, including non-human primates (NHPs) [4–13], cats [14], cattle [15], and horses [16], for most of which complete genomes are available [16–25]. Recently, a novel FV was discovered by metagenomics in a bat (*Rhinolophus affinis)* from China (RhiFV) and was partially sequenced [26]; however, whether RhiFV is bat-specific will require confirmation with population and epidemiological studies. Although virtually every NHP sampled has a species-specific FV, a human-specific FV has not been identified. Moreover, all FVs isolated from humans have been demonstrated to originate from zoonotic infections with simian FVs (SFVs) [27–36].

Similar to other retroviruses, FVs occasionally leave ‘fossils’ in host genomes, which are the relics of past infections. These genomic fossils are called endogenous retroviruses (ERVs) and result from germ line infections that are followed by vertical inheritance and thus are present in every cell of an infected organism. In contrast, exogenous retroviruses and hence exogenous FVs are transmitted horizontally from organism-to-organism via infectious body fluids and may be confined to certain cell types defined by their host tropism. Only two endogenous FVs have been discovered in mammalian genomes to date. One is present in the two-toed sloth (*Choloepus hoffmanni*) genome (SloEFV) which was found to have a genome organization characteristic of all contemporary FVs [37]. The second endogenous FV was found in the aye-aye (*Daubentonia madagascariensis)* genome (PSFVaye) which to date has only been partially characterized [38]. Given a broad range of FV mammalian hosts and the wealth of available whole mammalian genomes, the finding of only two endogenous FVs so far suggests that FV endogenization is a rare event. While a number of defective ERVs found in fish genomes have been reported to exhibit some similarity to known exogenous FVs [39–41], more evidence is required to definitively determine if these ERVs are truly endogenous FVs, or alternatively, are distinct lineages that branched early on in FV or retroviral evolution.

Owing to the availability of sequences from diverse mammals (Additional file 1: Table S1), coupled with the stable pattern of codivergence with their hosts, the evolutionary history of mammalian FVs can be studied in great depth. For example, phylogenetic analysis of extant SFVs revealed strong evidence of SFV-host co-speciation over more than 30 million years (Myr) [42]. The discovery and phylogenetic analysis of SloEFV [37] supported and extended the FV-host co-speciation hypothesis further to the basal radiation of eutherians which occurred more than 100 Myr ago (Ma) [43] and simultaneously expanded FV mammalian host range to cover xenarthrans, which are one of the four superorders of the Eutheria clade (Laurasiatheria, Euarchontoglires and Afrotheria are the other superorders). This is in contrast to lentiviruses, for which sequence availability is comparable to that of FVs, but their evolutionary history is not as well understood and much more difficult to investigate, as they do not always co-speciate with their hosts [44]. Nevertheless, at the present day, evidence of FV infection in, and sequence data from prosimians and afrotherian mammals is still lacking, and this is required to better understand the FV radiation and evolutionary history in eutherians. Here, we report the discovery and describe the first extant exogenous PSFV isolated from a lorisiforme galago (*Otolemur crassicaudatus panganiensis*) (PSFVgal) and a novel ERV, that is potentially an endogenous FV, present in the genome of an afrotherian Cape golden mole (*Chrysochloris asiatica*) (ChrEFV). We also further describe PSFVaye that was previously only partially characterized [38]. Together with the currently available FV sequences, we use detailed molecular analyses to re-examine the co-evolutionary history of mammalian hosts and their FVs.

## Results

### Characterization of the complete genome of the extant PSFVgal from a galago

**Figure 1:**
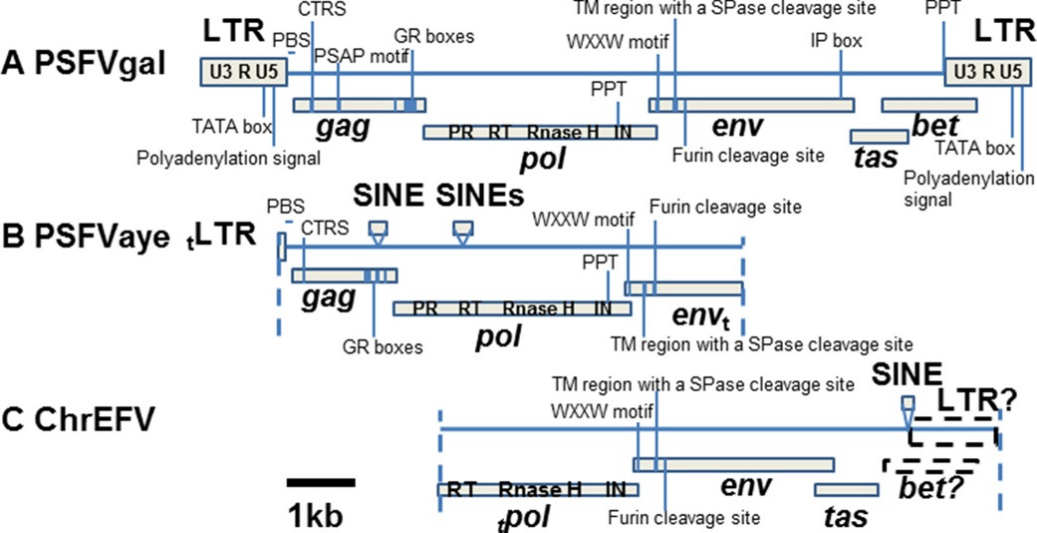
**Complete and partial genomic organizations of PSFVgal, PSFVaye and ChrEFV.** PSFVgal **(A)**, PSFVaye **(B)**, and ChrEFV **(C)** all exhibit at least some characteristic FV genomic features as indicated (see main text). PSFVaye and ChrEFV are interrupted by a few short interspersed nuclear elements (SINEs). Dashed lines indicate where genomes are truncated. Dotted boxes represent hypothetical domains which may be present but could not be identified. ‘t’-subscription indicates that the domain is truncated (5′-truncated: preceding the domain name; 3′-truncated: following the domain name). The scale bar (black solid line) represents a nucleotide length of 1 kb. LTR, long-terminal repeat; PBS, primer binding site; CTRS, cytoplasmic retention signal; GR boxes, glycine-arginine rich boxes; PR, protease; RT, reverse transcriptase; IN, integrase; PPT, polypurine tract; WXXW, conserved WXXW site; TM, transmembrane; SPase, signal-peptide peptidase; IP, internal promoter.

The complete genome of PSFVgal (GenBank accession number KM233624) was obtained by long-PCR amplification of overlapping 5′ and 3′ genomic halves using infected HeLa cells and sequence analysis. Genomic inspection shows that PSFVgal exhibits all the typical structural features of mammalian FV genomes, including the presence of *gag*, *pol*, *env*, *bel1*, and *bel2* genes flanked by LTRs (Figure 1), supporting that it is a FV. There are no in-frame stop codons identified in the PSFVgal coding regions. PCR and serological analyses also showed that PSFVgal-like sequences are not present in every tested Galago (7 sera and 10 DNA specimens) or Otolemur species (14 sera and 6 DNA specimens) (Additional file 1: Table S2) using a combination of PSFVgal-specific PCR (0/13), generic PSFVgal-PSFVaye PCR (6/16, 37.5%) and PSFVgal Western blot (WB) testing (16/21, 76%). Combined, these findings strongly support the notion that PSFVgal is an exogenous FV. The length of the complete genome is 12,118 nucleotides (nt). A detailed annotation of the PSFVgal genome is provided in Additional file 2: Figure S1.

Sequence similarity analysis revealed that the LTR length is 1,267 nt. An asparagine-1,2 tRNA-utilizing primer binding site (PBS) was identified downstream of the 5′-LTR before *gag* (TGGCGTCCCTGGGTGGGC, nt 1,270-1,287), but which is tRNA^Lys^ (TGGCGCCCAACGTGGGGG/C) in all other mammalian FVs. We confirmed the tRNA^Asp^ PBS sequences by population-based sequencing of multiple PCR products amplified on different dates using primers surrounding the PBS with two different primer pairs and two different concentrations (0.1 and 1.0 ug) of day 52 tissue PSFVgal-infected HeLa cell DNA. By comparison with the SFVmac LTR [17], the cap site defining the 5′ boundary of the R region was determined to be 20 nt downstream from the TATA box (TATATAA, nt 928–934) at nt 955 within the 5′-LTR, and the polyadenylation site within the 3′-LTR, which defines the 3′ boundary of the R region, was located at the CA dinucleotide (nt 11,955) 20 nt downstream from the polyadenylation signal (AATAAA, nt 11,930-11,935). Thus, the PSFVgal LTR U3, R, and U5 regions are 954, 150, and 163 nt long, respectively. Two polypurine tracts (PPT: AAGGAGAGGG) for the dual initiation of plus-stand DNA synthesis [45] were located at the 5′ boundary of the 3′-LTR (nt 10,842-10,853) and at the center of the genome toward the 3′ terminus of *pol* (nt 6,068-6,077) as anticipated.

The *gag* gene was predicted to be 1,932 nt long (nt 1,363-3,294) to generate a 644 amino acid (aa) protein. The cytoplasmic retention signal (CTRS), important for particle assembly and viral budding [46], was located at aa 73–90 (GNWGPGDRFARIEVLLRD). The position of a late-assembly domain proline rich PSAP motif, which is essential for primate FV particle assembly and budding [47, 48], was located at aa positions 217–220. The PSAP motif is highly conserved in all SFVs but its position within Gag is variable [48]. The three FV-specific glycine-arginine rich (GR I-III) motifs or boxes within the C-terminal region of Gag [49] were also determined (aa 479–493, 541–563, 567–597, respectively).

The Pol protein is 1,156 aa long (nt 3,248-6,718) and protease (PR), reverse transcriptase (RT), RNase H, and integrase (IN) were predicted to be located at aa positions 10–168, 154–355, 581–737, and 742–1,140, respectively. The catalytic centers of PR (DTG) and RT (YVDD) were located at aa 20–22 and 304–307, respectively. RNase H active sites were found to be at D^589^ and D^659^ and IN catalytic motif (DD35E) within the IN core domain (aa 862–972) was at D^926^-D^938^-E^962^.

The Env protein is 999 aa long (nt 6,603-9,602). We found a highly conserved WXXW motif (WLRW, aa 10–13) in the N-terminus which is essential for Gag-Env protein interaction during the budding process and is found in all known extant FVs [50, 51]. The internal promoter (TATAAAA) within the *env* gene, which serves for the initiation of *bel1* transcription, is present at nt 9,299-9,305. We also identified a hydrophobic transmembrane region, which contains a putative signal-peptide peptidase cleavage site (TMGWCIGLFCLLLILLFS↓LVIVIL), at aa 81–104. The cleavage occurs within the endoplasmic reticulum to remove the signal peptide [52]. A potential furin-cleavage site was also found (TRPNYTAARSRR↓SVE) at aa 131–145. It is this site at which the FV Env protein is cleaved, either by furin or furin-like proteases, to produce an Env leader protein – a component that interacts with the N-terminus of Gag via its WXXW motif and is absolutely required for FV particle budding [52].

The *bel1* and *bel2* genes were predicted to be present at nt 9,569-10,402 and nt 10,026-11,414, respectively. The putative Bel1 protein (277 aa) exhibits only a weak similarity (<15% identity) to extant FV Bel1 proteins, which could only be shown by comparison with Hidden Markov Model (HMM) of FV Bel1 proteins (HMM profile GyDB (http://www.gydb.org/): Bel1 spumaretroviridae; E = 3.7E-06). In contrast, the putative Bel2 protein (511 aa) exhibits a relatively high similarity with other FV Bel2 proteins with the best BLASTp score to the BFV Bel2 (E = 4E-08; maximum identity = 26%).

### Discovery and characterization of PSFVaye and ChrEFV

To search for endogenous FVs, we screened all publically available GenBank whole genome shotgun (WGS) sequences with various FV proteins (Additional file 1: Table S3) using tBLASTn. As previously reported [38], several matches were returned from the WGS assembled sequence of the aye-aye (*Daubentonia madagascariensis)* spanning four non-overlapping contigs (Table 1). Sequence analysis shows that these four contigs represent different parts of a single ERV ‘PSFVaye’, and this was also confirmed by genome walking. Between the first and the second, and the second and the third contigs are short interspersed nuclear elements (SINEs), and between the third and the fourth contigs are 5 ambiguous nucleotides. We found that PSFVaye contains numerous frameshift and in-frame stop codon mutations and is interrupted by several transposable elements throughout the genome, confirming that PSFVaye is endogenous and not replication competent. The notion that PSFVaye is endogenous was further supported by PCR testing of 18 different aye-ayes using PSFVaye *gag* primers in a single round of PCR (Additional file 1: Table S2); all of the aye-ayes including three infants were strongly PCR-positive which is typical of ERVs. Serums available from 17 of these aye-ayes were all negative for antibodies to the related PSFVgal (Additional file 1: Table S2), indicating they do not code for complete proteins, consistent with other evidence that PSFVaye is endogenous. This *in silico* screening process also returned one contig from the WGS assembled sequence from a Cape golden mole (*Chrysochloris asiatica*) (Table 1). We designated this element ‘ChrEFV’. Like PSFVaye, ChrEFV also contains numerous frameshift and in-frame stop codon mutations and is interrupted by several transposable elements, confirming that it is not replication competent as is typical of ancient ERVs. Genomic inspection revealed the putative genes of both PSFVaye and ChrEFV to be in the same order as those of typical exogenous FVs (Figure 1; see Additional file 2: Figure S1 for detailed sequence annotations). Furthermore, it is likely that there is only one copy of each virus present in either host genome since both the aye-aye and Cape golden mole genomes were extensively sequenced (38X [53] and 66X [54] coverage, respectively) but paralogs of each were not found.

**Table 1:** **Identification of foamy virus (FV)-like sequences in the aye-aye and Cape golden mole genome**

Accession no.	FV genomic region	Position*	BLASTp best hit	Query cover	Max identity	E-value
**Aye-aye (** ***Daubentonia madagascariensis*** **)**						
AGTM011839603	(5′ truncated) 5′-LTR	3208..3318	N/A	N/A	N/A	N/A
	Primer binding site	3321..3338	N/A	N/A	N/A	N/A
	*gag*	3405..4654	CAA70074 Gag FFV	93%	37%	6E-72
AGTM012070104		131..361				
	*pol*	323..1247	AAF64414 Pol EFV	95%	48%	0.0
AGTM011519752		90..1996 (end)				
AGTM010361255		1..635				
	(3′ truncated) *env*	520..2763	AFK85016 Env RhiFV	80%	42%	7E-115
**Cape golden mole (** ***Chrysochloris asiatica*** **)**						
AMDV01151999	(5′ truncated) *pol*	<615.. > 3534	AFX98084 Pol SFV	95%	47%	0.0
	*env*	<3464.. > 6371	AFR79245 Env BFV	96%	30%	5E-121
	bel1**	6338..6978	-	-	-	-

**‘**N/A’ = ‘Not applicable’.

*The nucleotide positions are with respect to the contig length and do not take insertion and deletion mutations into account.

**The similarity between ChrEFV Bel1 and other FV Bel1 proteins was determined by comparing the ChrEFV putative Bel1 protein against the Hidden Markov Model (HMM) of FV Bel1 proteins available in the GyDB (http://www.gydb.org/) (HMM profile: Bel1 spumaretroviridae; E: 0.24).

Within PSFVaye, via sequence homology, we identified i) a PBS (TGACACCCAATGTGGGGC**;** nt 114–131) with a broad tRNA usage (tRNA^Lys, Asn, Thr, Pro^) predicted, ii) a complete putative *gag* gene (nt 198–1,971) which is interrupted by a SINE (AluJo [55], nt 1,449-1,748), iii) a complete putative *pol* gene (nt 1,941-5,727; PR: nt 1,968-2,459; RT: nt 2,415-3,306; RNase H: nt 3,985-4,467; IN: nt 4,480-5,676) which is also interrupted by SINEs (AluJ Mim [56] & ALU [57], nt 2,871-3,165), and iv) a 3′-truncated putative *env* gene (nt 5,612-7,867). We also found ~113 nt on the 5′ end of the PBS to be uniquely-mapping within the aye-aye genome. This might represent some portion of the 5′ end of the 5′ PSFVaye LTR (nt 1–111). We confirmed the truncation of the putative 5′-LTR and *env* gene and the absence of a 3′-LTR in the PSFVaye genome by using a genome walking of genomic DNA from two different aye-ayes. We found that PSFVaye exhibits several characteristic FV features. For example, its Gag protein contains a degenerate, but still recognizable, putative CTRS (GPR?VGD*WQRICLAFQY, aa 37–54) and three GR I-III boxes (box I: nt 1,248-1,358; box II: nt 1,422-1,781 interrupted by a SINE (nt 1,449-1,748); box III nt 1,851-1,871); the putative *pol* gene harbors a PPT (AAGGACAAAG, nt 5,092-5,101); and the N-terminus of the putative Env contains a highly conserved WXXW motif (WLAW, aa 10–13). A hydrophobic transmembrane region containing a signal-peptide peptidase cleavage site (ILIWIMLFLILFSAILVS↓TLIAVF) was also located in the N-terminus of the putative Env at aa 63–86. We could not identify a furin-cleavage site in the Env of PSFVaye, most likely due to its defective nature. However, by comparing the Env sequence of PSFVaye to those of other exogenous non-defective FVs, we speculate that it should be located between aa 113 and 127 (YFSQAHV*KSRAIHF). Moreover, its putative Gag and Env proteins only exhibit similarities to FV proteins (Table 1) and share no detectible similarities with other (non-FV) retroviral proteins. All these findings strongly suggest that the PSFVaye sequence is an endogenous FV, and not merely a distantly related FV-like ERV.

ChrEFV contains i) a 5′-truncated putative Pol coding gene (nt <1- < 2,937) which only contains full RT (nt 1–597), RNase H (nt 1,270-1,737), and IN coding domains (nt 1,750-2,931), ii) a full putative Env coding gene (nt <2,867- > 5,785) and iii) a full putative Bel1 coding gene (nt 5,752-6,393). Following the putative *bel1* gene is a long non-repetitive sequence of ~1,700 nt containing a SINE (AFROSINE1B [58]). The *bel2* gene and the 3′-LTR of ChrEFV may be located within this region but cannot be identified, most likely due to divergence from the probes we used. Nonetheless, the N-terminus of the ChrEFV Env protein harbors the highly conserved WXXW motif (WMRW, aa 10–13) and only shows significant similarity to FV Env proteins (Table 1). A hydrophobic transmembrane region harboring a putative signal-peptide peptidase cleavage site (VMTSYVS?LILLGIIITA↓SFI TIC, aa 74–97) was also found in the putative Env at the anticipated location, and a potential furin-cleavage site (GQIISNSSSNRRKI) could be located at aa 125–138 within the putative Env as well. Furthermore, the putative Bel1 protein also exhibits some similarity to FV Bel1 proteins which could be demonstrated by using the HMM searching technique (HMM profile GyDB (http://www.gydb.org/): Bel1 spumaretroviridae; E = 0.24; note that this weak support is rather typical and expected given that this is a part of an endogenous FV and that FV Bel1 protein is not conserved among FVs [59]). Although a number of FV-specific genomic features as outlined in the PSFVgal characterization section could not be located, possibly due to the lack of sequence data and/or divergence, this genomic information supports a FV progenitor of ChrEFV.

**Figure 2:**
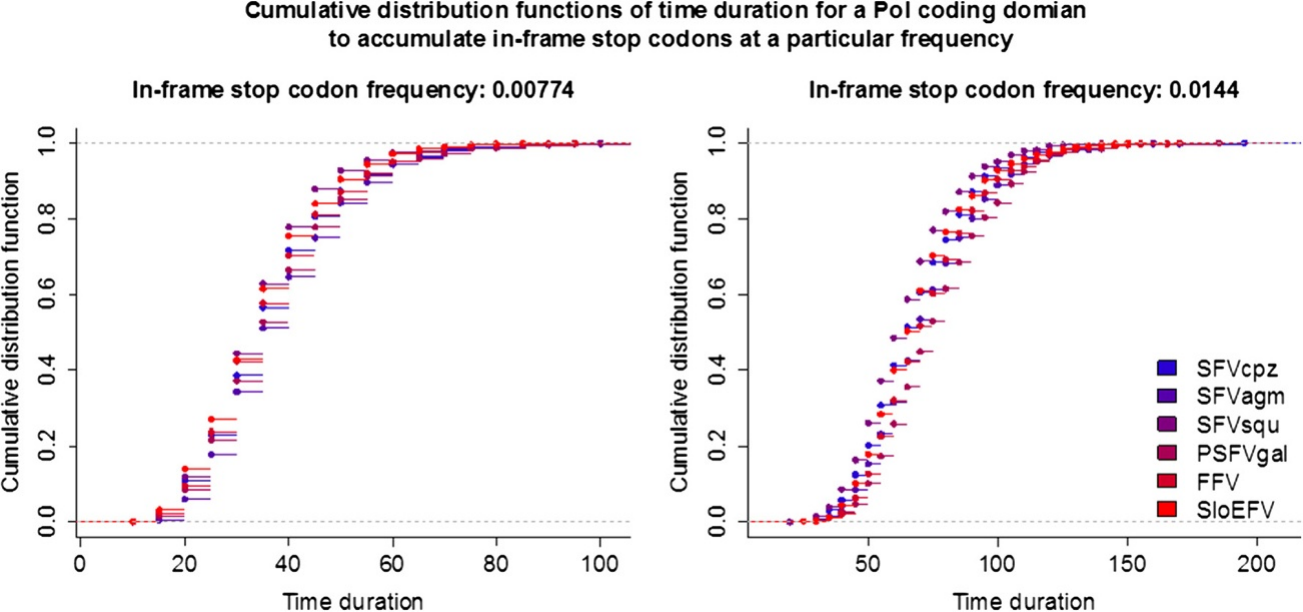
**PSFVaye and ChrEFV integration dates estimated from in-frame stop codon frequencies.** Cumulative distribution functions (CDFs) of time duration for Pols of SFVcpz, SFVagm, SFVsqu, PSFVgal, FFV, and SloEFV to accumulate in-frame stop codons at the frequencies of 0.00774 (right, stop codon frequency of PSFVaye Pol) and 0.0144 (left, stop codon frequency of ChrEFV Pol). Based on these CDFs, the mean age of PSFVaye is estimated to be ~35.2-39.6 millions of years (Myr) old (95% confidence interval: 15–75 Myr old, mode: 30–35 Myr old, median: 35 Myr old). The mean age estimate of ChrEFV is 65.6-78.2 Myr old (95% confidence interval: 35–125 Myr old, mode: 60–65 Myr old, median: 65–75 Myr old).

Aside from the aye-aye genome, currently available partial and complete genomes of other lemurs (*Lemur catta* and *Microcebus murinus,* respectively) do not seem to contain PSFVaye-like orthologs, confirmed by PCR testing of eight lemuriform species and one tarsiiforme species using generic PSFVaye and PSFVgal primers (Additional file 1: Table S2). These results support the hypothesis that endogenization of PSFVaye occurred after the basal diversification of the lemurs ~55-66 Ma [60–62], in the ancestral aye-aye lineage after it diverged from the ancestral lineage of all other living lemurs. Similarly, ChrEFV orthologs could not be found in elephant, hyrax, elephant shrew, and tenrec genomes, implying a maximum age of ~60-85 Myr based on the proposed Afrosoricida basal split date [43, 63]. To further estimate the age of these elements, we analyzed the frequencies of in-frame stop codons in their putative Pol proteins with a Monte Carlo simulation approach, using *pol*s of PSFVgal, SFVcpz, SFVagm, SFVsqu, FFV, and SloEFV (Figure 2) as model sequences. Consistently across all simulations, the mean age estimates of PSFVaye and ChrEFV are ~35.2-39.6 and ~65-78 Myr, respectively, congruent with (i.e. less than) their upper-bound age limit.

### FV phylogenetic analyses

A retrovirus phylogeny (Additional file 3: Figure S2) was estimated using an alignment of RT protein sequences to establish the phylogenetic positions of PSFVgal, PSFVaye, and ChrEFV among retroviruses. All three new FV sequences are placed with sequences of extant FVs. Together with the genomic characterization above, the phylogeny strongly supports the classification of PSFVgal as a true exogenous FV and that both PSFVaye and ChrEFV are real endogenous FVs. To the best of our knowledge, no exogenous lemuriform and afrotherian FVs have been identified to date. Thus, our finding of PSFVaye, and ChrEFV raises the possibility of the presence of undetected FVs circulating among other lemurs and afrotherians.

Interestingly, while we can confirm that PSFVaye and ChrEFV are endogenous FVs, a number of ERVs found in fish genomes, including the genomes of coelacanth (CoeEFV) [39], zebra fish (DrFV-1) [40], platyfish (platyfishEFV) [41], and cod (CodEFV) [41], have been reported to show some detectible similarity to exogenous FVs, but more evidence is required to definitively determine that these ERVs truly have a FV origin. Since these fish ERVs are phylogenetically basal to all known extant mammalian FVs, it is difficult to say with certainty that the progenitors of these ERVs are “true” FVs, and not distinct lineages that branched earlier in retroviral evolution but which are “FV-like” enough to be detected by sequence similarity using the true FVs as probes. Moreover, in terms of genomic organization, these fish ERVs do not show all the features characteristic of mammalian FV genomes. For example, the two identified accessory genes of CoeEFV show no significant similarity to those of extant mammalian FVs [39], only two out of five determined DrFV-1 proteins, Gag and Pol, are FV-like [40], FV-like accessory genes in platyfishEFV have not been located [41], and only FV-like RT could be found for CodEFV [41]. Although it is known that the accessory genes are the least conserved FV genes [59], and that this partial characterization of their genomes could be due to an ancient divergence of fish and mammalian FVs or simply the lack of sequence data, it raises a possibility that the progenitors of these ERVs may not be FVs but merely class III FV-like viruses. Resolution of this debate will require identification and analysis of FV genomes of other vertebrates like amphibians, reptiles, and birds, if they indeed exist [39]. Phylogenetic analysis of extant fish FVs and ERVs would also help resolve this debate.

**Figure 3:**
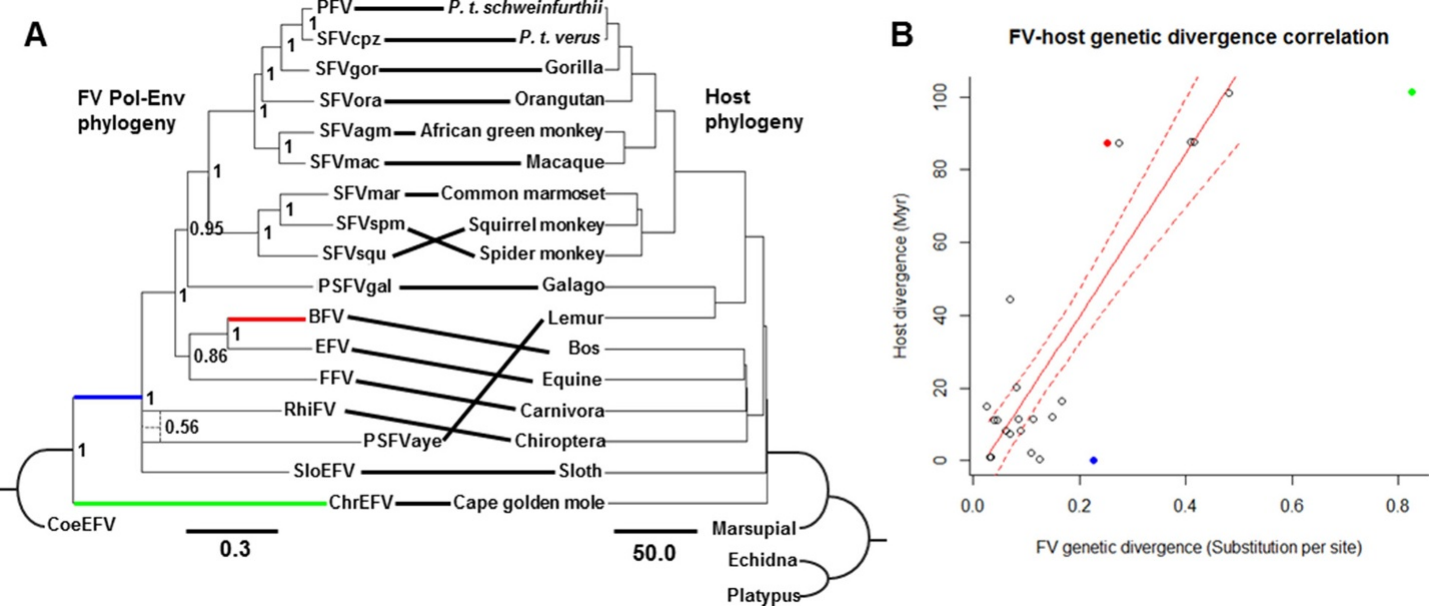
**Co-evolution of foamy viruses (FVs) and their mammalian hosts. (A)** FV Bayesian phylogeny (left) is compared to that of their hosts (right, previously published in [43]). Curved branches indicate outgroups. Numbers on branch nodes are Bayesian posterior probabilities. Solid lines between the two trees indicate FV-host associations. FV and host phylogeny scale bars are in units of amino-acid substitutions per site and million years, respectively. Weak support for the sister-taxon relationship of RhiFV and PSFVaye (dotted branches, posterior probability = 0.56) was inferred. Among 17 FV and host sequences, 3 apparent mismatches have been inferred using co-phylogenetic reconstruction. Branches corresponding to FV-host co-evolution were identified and used in FV-host divergence correlation analysis **(B)**. Branch lengths of FV tree and host divergence time were determined to have a linear correlation with a statistically strong support for its coefficient (linear regression: N = 23, R^2^ = 0.823, p = 3.48E-8), represented by the solid red line. Dotted lines represent the 95% confidence interval of the estimated linear relationship. Outliers determined by Cook’s distances (solid dots), including the bovine foamy virus (BFV) branch (red), the ChrEFV branch (green), and the ancestral branch leading to the exafroplacentalian FVs (blue), were not included in the linear regression. The colors of the outliers correspond to the colors of the branches in **A**.

To investigate FV phylogenetic relationships in more detail and evaluate the FV-host co-evolutionary history, we performed two analyses: (i) FV-host phylogenetic reconciliation analysis, and (ii) FV-host divergence correlation analysis. A Bayesian phylogeny of FVs was constructed based on an alignment of concatenated Pol-Env protein sequences and compared to the previously published host phylogeny [43] (Figure 3A). Gag protein sequences were not included in the alignment since (i) the Gag sequence of RhiFV is not available and (ii) the alignable region of Gag for the rest of the FVs is relatively short (~180 aa). Potential recombination among the sequences within the alignment was assessed using a quartet-based recombination detection program VisRD3 [64], and the results showed no significant evidence for recombination, both at nucleotide and protein levels (nucleotide: p = 0.621; protein: p = 0.495). Furthermore, we also found that neither of the separate Pol nor Env alignments reject the topology of the best phylogeny inferred from the concatenated Pol-Env alignment (Figure 3A), congruent with the results from the recombination test (approximately unbiased test: p-AU_Pol_ = 0.987; p-AU_Env_ = 0.700; Additional file 4: Figure S3). The two phylogenies show remarkably similar topologies, reflecting the well-established evolutionary history of stable FV co-speciation with their hosts [37, 42]. In total, 14/17 (82.4%) potential FV-host co-speciation events were inferred among the 17 FV and host sequences using the co-phylogeny reconstruction software Jane v4.0 [65]. The deepest co-speciation event occurred early on in eutherian diversification (Figure 3A) corresponding to the Exafroplacentalia-Afrotheria split. The reconstruction that maximizes the number of co-speciation events suggests that the most recent common ancestor (MRCA) of PSFVaye and RhiFV was present in the MRCA of their hosts and requires a duplication of the virus lineage at the base of the exafroplacentalian mammal clade (Additional file 5: Figure S4). Based on what we know about FV biology, it is extremely unlikely that a FV will diversify into two lineages within a single natural host, i.e. in the absence of host diversification. We therefore postulate that this viral lineage duplication represents an ancient host speciation event, where one of the resulting species is not sampled in our dataset. We therefore adopt a conservative approach and do not count the PSFVaye and RhiFV co-speciation event, constraining the number of co-speciation events in our reconstruction to 13 (76.5%). This estimate relies on the assumption of the existence of this un-sampled host lineage. Nevertheless, 13 co-speciation events are still greater than expected by chance (random tip mapping: sample size = 1000, p < 0.001), indicating that the FV-host co-speciation history is very stable. Moreover, there is a strong linear correlation between FV and host divergence which extends from the present to the exafroplacentalian/exafroplacentalian FV radiation (linear regression: N = 23, R^2^ = 0.823, p = 3.48E-8; Figure 3B), confirming previous estimates [37]. This result suggests that across this protracted time period the accumulation of FV sequence divergence occurred in proportion to host divergence. Combined, these findings strongly support stable FV co-divergence with their mammalian hosts for more than 100 Myr throughout the Cretaceous and Cenozoic eras as has been previously proposed [37].

Despite ChrEFV having an inferred co-speciation event (posterior probability = 1), the ChrEFV branch lengths, including both the terminal branch and the internal branch leading to other FVs, are longer than would be expected based on the FV-host divergence linear relationship (Figure 3B; identified as outliers, green and blue dots, respectively, with Cook’s distance investigation). Given that ChrEFV is basal to all known extant FVs, this finding challenges the notion that ChrEFV is an ERV that has a FV origin. Nevertheless, ChrEFV is a long terminal branch in the inferred FV phylogeny and has accumulated numerous neutral genetic changes since its ancient endogenisation. Coupled with long-branch attraction, this ‘pseudogene effect’ [66] could cause artificial inflation of the branch length with a concomitant deep placement of ChrEFV within the FV phylogeny. Thus, given the congruent ChrEFV/host phylogenies and the FV-like features of ChrEFV outlined above, it is still likely that ChrEFV is a genuine endogenous FV and not merely a class III FV-like ERV. Resolution of ChrEFV evolutionary history requires further analysis with other extant afrotherian, marsupial or monotreme FVs when and if they are identified. The results from these analyses could also elucidate the earlier events of mammalian FV diversification. To the best of our knowledge, this is the first (endogenous) afrotherian FV to be discovered, extending the FV host range to cover the whole eutherian clade.

PSFVgal was found to be a sister taxon of SFVs with robust support (posterior probability = 0.95), consistent with a FV-host co-speciation history. Additionally, we also identified a number of extant FVs from other lorises, including the silvery greater galago (*Otolemur monteiri monteiri*), southern lesser bush baby (*Galago senegalensis moholi*), and a potto (*Perodicticus potto*) (Additional file 1: Table S2), and partially sequenced their genomes (IN core domain: ~259 nt, 85 aa). When these new sequences were included in our phylogenetic analysis, they were all found to be distinct FVs forming a clade with PSFVgal with relatively high statistical support (posterior probability = 0.85) within which the branching orders generally mirror those of their lorisiforme hosts (Additional file 6: Figure S5). These results suggest that co-speciation between exogenous lorisiforme FVs and their hosts is very stable, extending to the species level. Together, our results indicate an ancient distribution and evolution of lorisiforme FVs in continental Africa dating to the divergence between strepsirrhine primates (lemurs and lorises) and the anthropoid primates about 70–95 Ma [43, 62, 63]. The absence of PSFV in some lorises (Additional file 1: Table S2) may be due to the testing of limited numbers of individuals and/or testing of captive animals that were captured as infants and thus were likely PSFV-negative at that time.

In contrast, PSFVaye was not a sister-taxon of PSFVgal as would be expected, showing a conflict in the co-evolutionary history of PSFVaye (Figure 3A). Instead, the sequence is robustly placed outside the boreoeutherian FV clade (posterior probability = 1), but still remains together with exafroplacentalian FVs (posterior probability = 1). The same evolutionary history was inferred for RhiFV (Figure 3A); instead of being grouped together with other fereungulata FVs (bovine, equine, and feline FVs) as would be expected if it were to co-speciate with its host, it is placed robustly outside the boreoeutherian FV clade (posterior probability = 1), but still remains together with exafroplacentalian FVs (posterior probability = 1). We compared our best estimated phylogeny against hypothetical alternative phylogenies where PSFVaye and/or RhiFV co-speciate with their hosts using approximately unbiased tests [67], and found that the co-speciation hypotheses of PSFVaye and RhiFV are both rejected (Additional file 4: Figure S3-A). Interestingly, our analysis also inferred a sister group relationship between PSFVaye and RhiFV although statistical support for this relationship is extremely weak (posterior probabilities = 0.56, Figure 3A).

## Discussion

Consistent with previous findings [37, 42], our analyses suggest an extremely stable FV-host co-speciation history across a timescale spanning millions of years. Interestingly, this is in contrast to what is suggested by various *in vitro* experiments. For example, an *in vitro* study has shown prototype FV (PFV) to be capable of infecting not only primate, but also rodent, laurasiatheria, avian and reptile cells, with an extremely broad range of susceptible cell types [68]. Furthermore, *in vitro* investigations of FV cell-attachment and entry also suggested that FVs of different species likely utilize the same, perhaps promiscuous, receptor molecule(s) for cell-attachment and/or entry [69–71]. Although these findings might in part help to explain FV cross-species transmissions (FV speciation events by means of jumping from one host species to another, without the host speciating at the time of the jump) observed in nature (see below), one would expect to see more host-switches happening given these findings. Analyses of several anti-retroviral restriction factors have shown that they are specific to particular FVs. For example, while the tripartite motif protein 5αs (TRIM5αs) of most New World monkeys (NWMs) have been shown to be able to restrict some NWM FVs, PFV and a SFV from macaque, TRIM5αs from apes cannot, but instead can restrict feline FV [19, 72]. Inhibition of apolipoprotein B mRNA-editing, enzyme-catalytic, polypeptide-like 3C (APOBEC3C) by FVs has also been shown to be species-specific [73]. Whether this species-specific FV-host antagonistic interaction is one of the factors underlining the stable co-speciation history or the result of it is still unclear. To date, the factors that determine this extremely stable pattern of FV-host co-speciation in nature are still very poorly understood.

**Figure 4:**
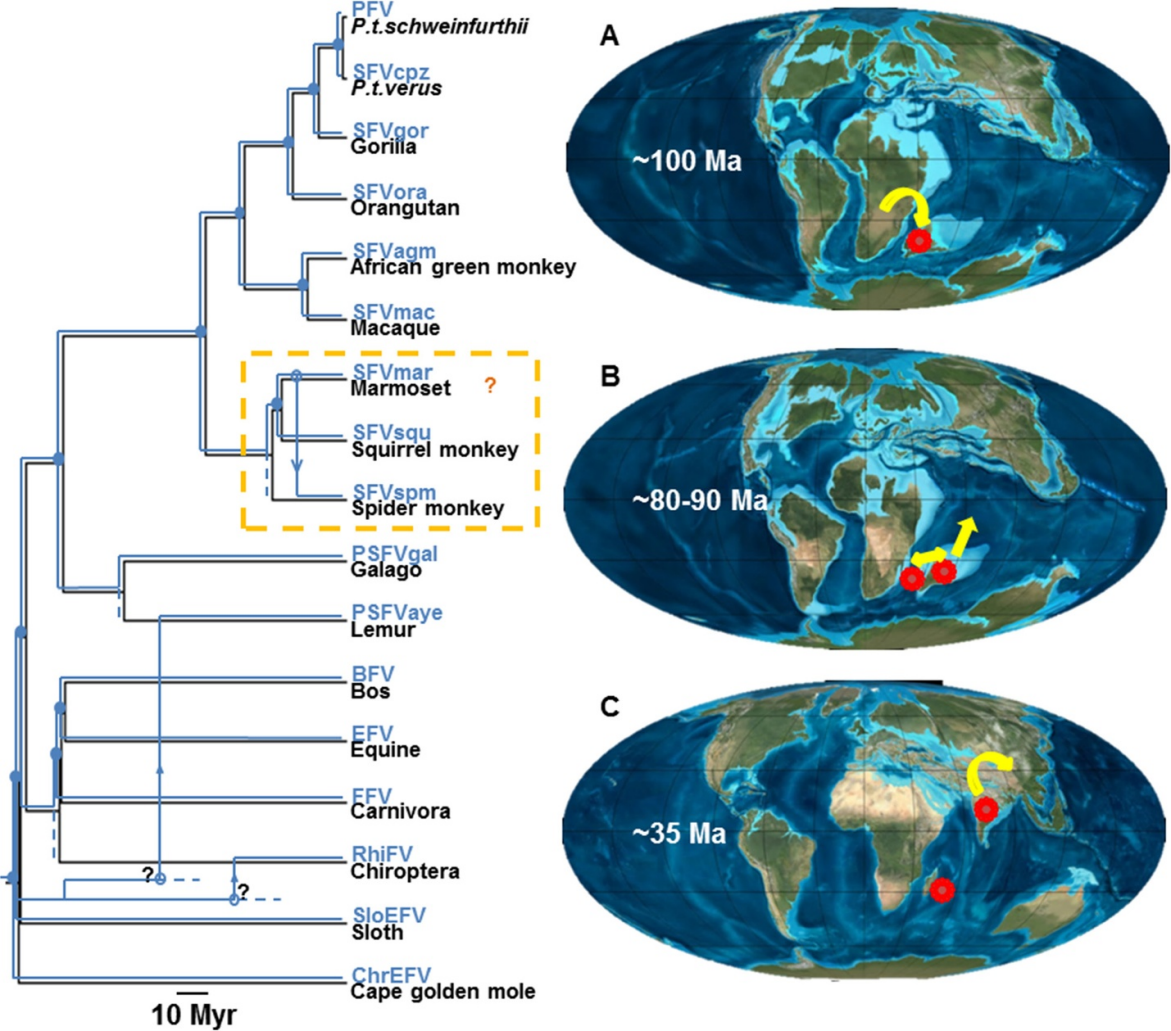
**Hypothesis explaining the mismatches in foamy virus (FV)-host evolutionary history.** FV phylogeny (blue) was superimposed upon host phylogeny (black) and is scaled to host divergence times. The scale bar is in units of millions of years. Solid circles represent FV-host co-speciation events. Open-circles represent possible host switching events of which the inferred switching directions are indicated by arrows. A hypothetical scenario of host switching (as indicated by an orange ‘**?**’) within the New World monkey FV clade is shown in an orange box. An alternative scenario is shown in Additional file 5: Figure S4. For PSFVaye and RhiFV, we speculate that, in the very early history of mammalian evolution, eutherians came to the Madagascar-India landmass with their FVs (red circles) upon which they established stable populations **(A)**. The landmass was then split into the Madagascar and India landmasses resulting in the FV population splitting into two separate groups ~80-90 Ma **(B)**; one FV variant remained on Madagascar and the other was transported to Laurasia via the Indian landmass continental drift **(C)**. The former gave rise to the PSFVaye progenitor while the latter gave rise to RhiFV. Directions of infected FV host population movements are indicated by yellow arrows. However, it is unknown which ancestral species introduced FV to the lemur and bat lineages and when the transmissions occurred (as indicated by a black ‘**?**’). Additional sequence data from other mammals, especially bats and lemurs, are required to further resolve these aspects of FV evolutionary history.

Although we found the pattern of FV-host co-speciation to be very strong and stable, it is not absolute. Against a clear background of FV-host co-divergence are a small number of mismatches in FV-host evolutionary history. One involves NWM FVs which form a clade that is sister to catarrhine primate FVs, reflecting the branching order of their hosts. However, the branching orders within the NWM FV clade clearly do not parallel that of their platyrrhine hosts [60, 74, 75] (Figure 4), with SFVmar from a common marmoset being more closely related to SFVspm from a spider monkey than to SFVsqu from a squirrel monkey, as would be expected under a co-evolutionary scenario. We compared our inferred phylogeny against an alternative phylogeny in which NWM FVs co-speciate with their hosts using approximately unbiased tests [67], and found that the co-speciation picture is rejected (Additional file 4: Figure S3-B). These results are consistent with results from a previous study that used an alignment of Gag protein sequences [19] and implied an ancestral NWM FV host switch (Figure 4 and Additional file 5: Figure S4, orange boxes). Marmosets, squirrel monkeys, and spider monkeys are closely related, occupy large overlapping geographic ranges [19], and are commonly used in biomedical research. It is thus plausible to imagine a scenario under which heterologous NWM FV infections might have occurred in the past, either in the wild or during captivity. This is unsurprising given that this type of FV cross-species transmission between closely related primate species has already been documented [30]. In contrast to our results, a phylogenetic analysis of a wider range of NWM FVs using short *pol* nucleotide sequences (276 nt) suggested that these three NWM FVs co-speciate with their hosts and that host switches occurred elsewhere [76]. Analysis of additional NWM FV complete genomes will be necessary to distinguish these possibilities.

A more extreme case of host switching across taxonomic orders may explain the phylogenetic placement of PSFVaye and RhiFV, where both are placed robustly outside the boreoeutherian FV clade (posterior probability = 1). Nevertheless, one possibility for the inferred placement of PSFVaye may be that it is an artefact due to neutral genetic changes that have accumulated since its endogenization [38]. To examine whether or not neutral evolution alone could be responsible for the observation, Bayesian phylogenies of concatenated Pol-Env protein alignments containing artificially and uniformly mutated SFVagm, SFVsqu, and PSFVgal sequences were inferred. These sequences were mutated *in silico* with an overestimate of the mammalian neutral substitution rate of 10E-9 substitutions per site per year (s/n/y) over a mean estimate of PSFVaye endogenization date of 40 Myr ago (Figure 2). The substitution process was assumed to be neutral, homogenous, and independent across sites and base types. Re-aligning the sequences after the simulation using MUSCLE implemented in MEGA6 [77] does not change the relative position of mutated sequences in other SFV sequences. Our results suggest that, even with this biased substitution rate setting of 10E-9 s/n/y, the neutral genetic change accumulation alone is insufficient to explain the inferred phylogenetic position of PSFVaye. Our *in silico* analysis only increased the branch length and decreased the branch support without changing the tree topology (Additional file 7: Figure S6). It is thus unlikely that the placement of PSFVaye is an artefact. Similarly, the placement of RhiFV is also likely to be genuine since it is an extant exogenous FV, supported by the absence of in-frame stop codons or frameshift mutations, and that it was found to be present in viral particles and not host genomic material [26]. In addition, the RhiFV lineage remains in the same phylogenetic position even after removal of PSFVaye from the phylogenetic analysis.

We then asked whether there is a reasonable FV host switching model that can explain both (i) the deep phylogenetic placement of PSFVaye and RhiFV which is basal to all known exafroplacentalian FVs, and (ii) the dispersed geographical distribution of their hosts (Madagascar and China, respectively). We propose the following scenario to explain the phylogenetic relationships of PSFVaye and RhiFV within the context of the evolutionary and geographical history of FV hosts (Figure 4). Approximately 180–160 Ma, Gondwana was split into two separate landmasses: the Madagascar-India-Antarctica (MIA) landmass and the Africa-South America (ASA) landmass [78, 79]. The MIA landmass then split into the Madagascar-India and the Antarctica landmasses about 135 Ma [78, 79]. Thereafter, but still in the very early period of eutherian evolutionary history, eutherians arrived onto the Madagascar-India landmass upon which their FVs established stable populations (Figure 4A). Since then, these early eutherian FVs evolved independently from, and hence have distant evolutionary relationships with, FVs circulating on the African landmass, which later would give rise to all extant SFVs, lorisiforme FVs and fereungulata FVs. The India landmass was then split from the Madagascar landmass about 80–90 Ma [78, 79] (Figure 4B), separating the FV populations into two groups: one circulating in Madagascar which gave rise to the progenitor of PSFVaye and the second transferred to Laurasia via the India landmass continental drift which independently gave rise to RhiFV (Figure 4C). However, the model does not suggest which ancestral species introduced FV to the lemur and bat lineages and when these transmissions occurred. Nonetheless, both PSFVaye and RhiFV are placed robustly with exafroplacentalian FVs but outside the boreoeutherian FV clade (posterior probability = 1 and 1, respectively). This suggests that the original hosts were exafroplacentalians, and not boreoeutherians, and that the host switches occurred between species across deep clades long diverged from each other. Sequence data of FVs from additional mammals, especially bats and lemurs, are required to examine further the evolutionary histories of PSFVaye and RhiFV.

Our hypothesis helps to explain the deep phylogenetic placement and dispersed geographical distribution of PSFVaye and RhiFV. In addition, this hypothesis also gives an explicit prediction that PSFVaye and RhiFV should share a MRCA prior to 80–90 Ma based on the estimated Madagascar-India landmass split date [78, 79]. Indeed, although not well-supported (posterior probability = 0.56), our best phylogenetic estimate suggests that PSFVaye and RhiFV have a sister-group relationship (Figure 3A). Assuming that our inferred PSFVaye-RhiFV relationship is correct, we estimated the tMRCA of PSFVaye and RhiFV to be 93 (85–101) Myr based on the linear relationship of FV-host diversity (Figure 3B). Although slightly high, our estimate range largely overlaps with the date range derived from this hypothesis. The accumulated neutral changes in the PSFVaye sequences may explain both the weak branch support and the slightly overestimated divergence date.

## Conclusions

Here, we report the characterization of the complete PSFVgal genome obtained from a galago, and describe in more detail the partial genome of PSFVaye from the aye-aye. The genomic organization of PSFVgal and PSFaye is characteristic of FVs, and they are phylogenetically placed within the FVs. The defective nature of the PSFVaye genome, coupled with its robust amplification from genomic DNA and an absence of antibodies to the genetically related PSFVgal in infected animals, indicates that it is endogenous. In contrast, PSFVgal has a complete viral transcriptome, elicits a FV-specific immune response, and is not present in all individuals, consistent with other exogenous FVs. We also report the discovery and describe a novel ERV present in the Cape golden mole genome, ChrEFV. Genomic inspection and analysis suggests that ChrEFV is also an endogenous FV, and its phylogenetic placement is consistent with a history of co-speciation between FVs and their mammalian hosts. However, ChrEFV has a high level of accumulated sequence divergence compared to other FVs, likely an artefact of relaxation of evolutionary constraints since becoming endogenous.

We found a general, stable pattern of mammalian FV-host co-divergence which extends as deep as the exafroplacentalian basal diversification, spanning more than 100 Myr. To date, it is still poorly understood why FVs stably co-speciate with their natural hosts. Furthermore, we also identified two possible cases of host switching in the evolutionary history of FVs. The first was observed in NWMs, as previously shown [19], which may have happened during captivity or in the wild and which has previously been reported to rarely occur in Old World monkeys and apes [30]. However, others have shown congruent NWM and SFV phylogenies using a larger distribution of species-specific sequences [76]. The second involves PSFVaye and RhiFV which may involve cross-species transmission at the level of mammalian orders. We propose a scenario based upon geographical knowledge of continental drift and hypothetical migration of ancient eutherians to explain this observation. Our results highlight the value of integrating multiple sources of information to elucidate the evolutionary history of mammals and their viruses, including continental and geographical histories, ancestral host locations, in addition to the natural history of host and virus.

## Methods

### Nonhuman primate (NHP) samples and nucleic acid preparation

NHP specimens (serum, frozen or fresh whole blood, tissues) were purchased from the Duke Lemur Center, were collected on an opportunistic basis from seven U.S. zoos following approved animal use protocols, or dried blood spots (DBS) via NHP hunters in Cameroon from freshly hunted NHP bushmeat in a study approved by Institutional Animal Care and Use Committees and the Cameroon government (Additional file 1: Table S2). Hunters were educated about the risks associated with direct contact with NHPs and about appropriate prevention measures. Prior to processing, all specimens were stored at -20°C or -80°C, except DBS which were stored in nytran ziplock bags with dessicant at room temperature. DNA lysates or nucleic acids were prepared from blood specimens and DBS as previously described [28]. Nucleic acids were extracted from archived tissue specimens from the Duke Lemur Center (liver, spleen, kidney) using a Qiagen QIAmp DNA mini kit. DNA concentrations were determined using a Nanodrop spectrophotometer and DNA integrity was confirmed with ß-actin PCR.

### Isolation of PSFVgal

HeLa cells were infected with PSFVgal (SFV-5; ATCC# VR-644), originally isolated from the throat swab of an *Otolemur crassicaudatus panganiensis*, and grown in complete DMEM medium supplemented with 10% FBS, 2 mM L-glutamine, 100 ug/ml streptomycin and 100 U/ml penicillin (Invitrogen). PSFVgal is the only viral isolate analyzed in our study. Cell cultures were incubated at 37°C and 5% CO2 and were split when 90% confluent. Cellular DNA was prepared using a Qiagen kit and quantified with a Nanodrop spectrophotometer when cytopathic effect was observed in >50% of the cells.

### Western blot (WB) assay

PSFVgal-infected and uninfected cells were pelleted by centrifugation at 1,500 rpm for 10 min and washed 2X with phosphate-buffered saline. Antigen for WB testing was prepared by treating cell pellets with WB sample buffer (Invitrogen) at 100°C for 10 min. Protein concentrations were determined using the BioRad DC Protein Assay kit and 150 ug of infected and uninfected HeLa cell lysates were applied separately to 4-12% polyacrylamide gels (Invitrogen) for WB analysis. Serum specimens were tested using a 1:50 dilution as previously described [80].

### PCR-amplification of 5′ and 3′ PSFVgal genome halves, plasmid cloning, and sequence analysis

To obtain the full-length genomic sequence of PSFVgal we first PCR-amplified small regions in the LTR and polymerase (*pol*) gene by using nested PCR with degenerate SFV primers and conditions provided elsewhere [80, 81]. PSFVgal-specific primers were then designed from the LTR and *pol* fragments to amplify two overlapping genomic halves using PCR and a Roche Expand 20 kb PCR System Kit following the manufacturer’s instructions. The Expand kit contains thermostable Taq DNA polymerase and a thermostable DNA polymerase with proofreading activity to minimize PCR-induced sequence artefacts. Briefly, the PCR reaction mixture was prepared in 50 ul reaction volumes containing 500 uM of each dNTP, 400 nM of each forward and reverse primer, and 5 units of Expand 20 kb enzyme mix using 0.5 ug of PSFVgal tissue culture DNA. The LTR-*pol* fragment was amplified using the primers PSFVgal-LF1 5′ GGC TTG GAT AAT TAA TTG TTA GAT GCT CTG 3′ and PSFVgalpolF2R 5′ GTT CCA AAC GTA TGC CCC TCT CCT T 3′. The PSFVgal *pol*-LTR fragment primers are PSFVgalpolF1 5′ GTC AGC ATT CAC CTC TTC CAC CTT G 3′ and PSFVGAL-LR1 5′ GAC TTA TTT ATT ACT GCA AGA CCC GAG AGG G 3′. Following denaturation at 92°C for 2 min, amplification consisted of 10 cycles at 92°C for 10 secs, 60°C for 30 secs, and 68°C for 6 mins, followed by 20 cycles at 92°C for 10 secs, 60°C for 30 secs, and 68°C for 6 mins with an additional 10 sec per cycle.

PCR products were visualized with 0.8% agarose gel electrophoresis and amplicons of the expected size were collected using a QIAGEN gel purification kit. Purified amplicons were cloned into the pCR-XL-TOPO vector (Invitrogen) for genome sequencing. Two plasmids were obtained; pCR-XL-TOPO- PSFVgal-LP containing the LTR-*pol* insert (5,148-bp) and pCR-XL-TOPO- PSFVgal-PL containing the *pol*-LTR insert (6,201-bp). The PSFVgal plasmid DNAs were purified using the Purelink Hipure Plasmid Filter Purification Kit (Invitrogen) and sequenced in both directions using a 3130XL Genetic Analyzer (Applied Biosystems). Complete genomes were assembled using the software program Vector NTI v11.1. 5′ and 3′ LTRs were determined based on overlapping sequences and comparison with available FV genome sequences.

Homology of PBS sequences to tRNAs was inferred using the transfer RNA database (http://trnadb.bioinf.uni-leipzig.de/). Confirmation of the tRNA PBS sequence was done by PCR amplification with primers flanking that region and using 0.1 and 1.0 ug of PSFVgal-infected HeLa tissue culture DNA. Primary and nested primers were used to independently amplify two products of different lengths and amplification was done separate from that to obtain the 5′ halve of the genome containing the PBS. Primary PCR primer sequences are F982 (5′-GAC CAG TGT GAG ATT GGT GTC TC-3′) and R1547 (5′-CCA GTT GCC TCC TGT GAT TCT AAC-3′) and the internal primers are F1133 (5′-CTC CTG GTT GAG GAC AAG GGA AC-3′) and R1475 (5′-CCA ATT TGT GCT CGT GGC ACT GG-3′) and standard PCR conditions were used. Numbers in the primer names reference their locations in the PSFVgal genome. PCR products from both primary and nested and 0.1 and 1.0 ug DNA inputs were sequenced.

### Discovery and characterization of ChrEFV and PSFVaye

Publically available GenBank whole genome shotgun (WGS) sequences were screened using tBLASTn and various FV protein sequences (Additional file 1: Table S3). As previously reported by Han and Worobey [38], several matches were returned from the WGS assembled sequence of the aye-aye, *Daubentonia madagascariensis,* spanning four non-overlapping contigs. The adjacency of these four contigs and sequences flanking the *gag* and *env* regions, especially the LTR and host integration sequences, were determined using a Seegene genome walking kit (Seegene, MD, USA). Three rounds of PCR were performed using universal primers provided in the kit (DW2-ACP, DW2-ACPN, UNIP2) with PSFVaye-specific primers (1st PCR primers: upstream PSFVAYE-1174R/DW2-ACP; downstream, PSFVAYE-6122 F/DW2-ACP; 2nd PCR primers: upstream, PSAGF2REV/DW2-ACPN; downstream, PSAER2FOR/DW2-ACPN; 3rd PCR primers: upstream, PSAGF1REV/UniP2; downstream, PSAER1FOR/UniP2). Positive bands were cloned and Sanger sequenced using an ABI7700 instrument. Furthermore, this *in silico* screening process also returned one contig from the WGS assembled sequence of a Cape golden mole (*Chrysochloris asiatica*), designated ‘ChrEFV’ here. Both were annotated via sequence homology. The presence of simple repetitive elements was determined by CENSOR (http://www.girinst.org/censor/).

### PCR testing of Prosimian genomic DNA specimens for FV

1 ug of prosimian DNA was PCR tested for FV *pol* sequences using a combination of nested primer sets or a single round of PCR to detect PSFVaye *gag* sequences depending on the species (Additional file 1: Table S2). The first PCR assay is PSFVgal-specific and is based on the PSFVgal genome and uses the first and second round PCR primers SIF1GAL 5′ CTT GCT GTG CAG AGC AGT CAC AAG GT 3′ and SIR1GAL 5′ GTT TTA TTT CAC TGT TTT TCC GTT CCA C 3′ and SIF3GAL 5′ CCA AGT CTG GAT GCA GAG CTT ATC CA 3′ and SIR3GAL 5′ ACT TTG GGG GTG ATA CGG AGT ACT 3′ to generate 712-bp and 635-bp fragments, respectively. The second assay was designed using an alignment of complete Old World and New World primate SFV genomes and uses the primary primers SIF5N 5′ TAC ATG GTT ATA CCC CAC KAA GGC TCC TCC 3′ and SIR5N 5′ AAT AAW GGA TAC CAC TTT GTA GGT CTT CC 3′ and nested primers SIP4N 5′ TGC ATT CCG ATC AAG GAT CAG CAT T 3′ and SIR1NN 5′ GTT TTA TYT CCY TGT TTT TCC TYT CCA CCA T 3′ to generate to generate 282-bp and 141-bp *pol* sequences, respectively. The third assay was designed using an alignment of PSFVgal and PSFVaye genomes and uses the primary primers 3′ FVPF05 5′ KKM TAY TGG TGR CCT AAT ATG 3′ and FVPFR5 5′ GGT CTW CCA ACY ART AGT TTA G 3′ and nested primers FVPF01 5′ CCT TTT GAT AAA ATY TAT ATG G 3′ and FVPR01 5′ CAS CTT TCC ACT ACT TTG G 3′ to generate 483-bp and 292-bp products, respectively. To detect PSFVaye *gag* sequences the primers PSAGF1 5′ AAG ACC CTT GCT GCC TAA TGT TGG 3′ and PSAGR1 5′ TAT TTG TAA CCA GGG CTT GAC CAG 3′ were used to amplify a 475-bp sequence. 5 ul of primary PCR product was used as template in the nested PCR reaction. Selected PCR products were visualized by agarose gel electrophoresis analysis, extracted using a QIAquick Gel Extraction kit, and sequenced in both directions using an ABI7700 instrument.

### Estimating the integration dates of PSFVaye and ChrEFV with Monte Carlo simulation

To estimate the age of PSFVaye and ChrEFV, we analyzed the frequencies of in-frame stop-codons in their Pol protein sequences. Excluding a hypothetical last stop codon, PSFVaye (1,163 codons) and ChrEFV (975 codons) Pols contain 9 and 14 in-frame stop codon mutations, which are equivalent to stop codon frequencies of 0.00774 and 0.0144, respectively. Using a Monte Carlo simulation approach and *pol*s of PSFVgal, SFVcpz, SFVagm, SFVsqu, FFV, and SloEFV as model sequences, we estimated two cumulative distribution functions (CDFs) of time duration for in-frame stop codons to be accumulated at these two frequencies for each model sequence. Each *pol* model sequence was mutated *in silico* over various hypothetical time durations from 5 Myr to 200 Myr with an increment of 5 Myr. The mutation (substitution) process was assumed to be neutral, homogenous, and independent across all sites and base types with the rate of 2.2E-9 s/n/y, an average substitution rate of mammalian genomes [82]. 1,000 simulations were performed for each period of time and the frequency of in-frame stop codons was then calculated for each simulation. Two separate CDFs of time duration were constructed for each sequence model using stop-codon frequencies of 0.00774 and 0.0144. In total, we constructed six CDFs for each of the two stop-codon frequencies. Means, medians, modes and 95% confidence intervals of the time duration for in-frame stop codons to accumulate at the two frequencies were calculated.

### Phylogenetic analysis

To investigate the phylogenetic relationships of PSFVgal, PSFVaye, and ChrEFV with other retroviruses, a consensus unrooted phylogenetic tree was constructed using a manually curated alignment of 240 RT protein sequences (162 aa) from alpha-, beta-, gamma-, delta-, epsilon-, lenti-, and spuma-like retroviruses using RAxML 7.2.8-HPC2 on XSEDE [83]. The alignment is available from the authors upon request. The LG + G + F model was determined to fit the data best by using ProtTest 2.4 [84]. Bootstrap support values for the branching order were calculated using 5,000 pseudoreplicates.

To further investigate the phylogenetic relationships of PSFVgal, PSFVaye, and ChrEFV with other FVs, a Bayesian FV phylogeny was estimated using MrBayes 3.2.1 [85] and a manually-curated alignment of concatenated Pol-Env sequences (893 and 603 amino acids, respectively). We used CoeEFV [39] to root our mammalian FV tree since it is the most immediate outgroup of mammalian FVs known to date although it is still debatable whether this ERV is a real endogenous FV or not. Gag sequences were not included in the alignment since (i) the Gag of RhiFV is not available and (ii) the alignable region of Gag for the rest of the FVs is relatively short (~180 aa). Potential recombination among the sequences within the alignment were checked using VisRD3 [64] both at nucleotide and protein levels. For both analyses, the null distribution was built based on 1,000 sets of randomly-shuffled sequences and the extended statistical geometry (Hamming) weighting option was selected. In the nucleotide analysis, the window size and step size was 300 and 60 nt, respectively; while in the protein analysis, the window size and step size was 100 and 20 aa, respectively. The results showed no significant evidence for recombination. The alignment is available from the authors upon request. The protein phylogeny reconstruction was performed using best available protein-specific model partitions of rtREV + Γ(4) + F (Pol) and WAG + I + Γ(4) + F (Env) as determined by ProtTest 2.4 [84] under the AIC criterion. The MCMC was run for 5,000,000 steps with the initial 25% discarded as burn-in. Trees and parameters were logged every 100 steps. Parameter value convergence and sampling independency were manually inspected and had effective sample sizes >1,000.

We evaluated the FV-host co-speciation hypothesis using two analyses: (i) FV-host phylogenetic reconciliation analysis and (ii) FV-host divergence correlation analysis. Phylogenetic reconciliation analysis of FV and host trees was performed using Jane v4.0 [65] (Genetic algorithm: number of generations = 100, population size = 100). The vertex-based cost mode was used, and the costs were set as follows: co-speciation = -1, duplication = 0, duplication & host switch = 0, loss = 0, and failure to diverge = 0. This setting was adopted in order to maximize the number of co-speciation events. The probability of observing the inferred number of potential co-speciation events by chance was calculated by random tip mapping in Jane v4.0 [65] (Genetic algorithm: number of generation = 100, population size = 100, sample size = 1,000). This method of analyzing phylogenetic incongruence cannot formally differentially weight transmission at different taxonomic levels however, and the derivation of p-values relies upon the interchangeability of branches. Thus, it does not discriminate between distant and close interspecies transmissions. Once the potential FV-host co-speciation events were located, we then identified FV-host corresponding branches that represent FV-host co-evolutionary histories for the FV-host divergence correlation analysis. In this test, we investigated whether or not the lengths of these corresponding branches were linearly correlated, i.e. whether or not the accumulation of FV sequence divergence occurred in proportion to host divergence, using linear regression implemented in MATLAB [86]. Outliers were determined by using Cook’s distance inspection. Outliers were removed one at a time using a threshold value of 3x mean of the observed Cook’s distances and the model was re-fitted until no outliers were found. The model was then used to estimate how long the parental branch of RhiFV and PSFVaye branches (0.0564 amino-acid substitutions per site) is in units of time. Given that the radiation of exafroplacentalians was 101.1 Ma [43], we then re-calculated when PSFVaye and RhiFV shared a MRCA to be 93.342 (85.095-101.589) Ma.

Our inferred phylogeny suggests two possible cases of FV host-switches: one involves PSFVaye and RhiFV and the other involves NWM FVs. Approximate unbiased (AU) tests using multi-scale bootstrapping [67] were performed to investigate whether or not our Pol-Env/Pol/Env alignments reject the co-speciation model for PSFVaye and RhiFV, and NWM FVs (Additional file 4: Figure S3). Four completing alternative phylogenetic placements of PSFVaye and RhiFV (Additional file 4: Figure S3-A), and two alternative placements of NWM FVs (Additional file 4: Figure S3-B) are compared to each other. The site-wise log likelihoods used in the tests were computed using PAML 4.7a [87]. The best available amino acid substitution model, as determined by ProtTest 2.4 [84] under the AIC criterion, was used (Pol: rtREV + Γ(4) + F; Env: WAG + Γ(4) + F). A molecular clock was not imposed, and ambiguous sites were included. AU tests were performed in Consel [88] with default settings.

Additional Bayesian FV phylogenies were constructed using the same settings as described above. One included a number of short fragments of other lorisiforme FV Pol sequences (IN core domain sequences, 85 aa) to further investigate phylogenetic relationships of lorisiforme FVs with other FVs. The additional Bayesian phylogenies included several *in silico* mutated sequences to examine the effects of neutral genetic changes on the inferred phylogenetic relationships. The alignment was not re-aligned after the sequence simulation (see main text). Both alignments are available from the authors upon request.

## Electronic supplementary material

Additional file 1: Table S1: Mammalian foamy viruses. **Table S2.** Distribution of simian foamy virus in prosimians. **Table S3.** GenBank accession numbers of protein sequences used as probes to search for integrated mammalian foamy viruses (FVs) as well as for phylogenetic analyses. (DOCX 63 KB)

Additional file 2: Figure S1: Detailed descriptions of PSFVgal, PSFVaye, and ChrEFV complete or partial genomes. Protein open reading frame (ORF) and putative reading frame (RF) locations were determined by sequence similarity to other FVs and by searching against the GyDB (http://www.gydb.org/). Short interspersed nuclear elements (SINEs) were determined by CENSOR (http://www.girinst.org/censor/). TATA boxes, polyadenylation signals, primer binding sites (PBS), and polypurine tracts (PPT) are indicated by bold type and solid lines. Long terminal repeats (LTRs), Gag, Pol, Env, Bel1, and Bel2 proteins are highlighted in grey, green, purple, blue, orange, and red, respectively. Several conserved domains and catalytic centers are highlighted in darker colors. Putative insertion and deletion mutations are indicated by red dashes. The beginning and the end of ORFs and RFs are indicated by arrows and square-arrows, respectively. (PDF 386 KB)

Additional file 3: Figure S2: A consensus unrooted retrovirus reverse transcriptase phylogeny. The phylogeny was built using an alignment of reverse transcriptase proteins of several retrovirus genera: alpharetrovirus (α, light green), betaretrovirus (β, dark green), gammaretrovirus (γ, blue), deltaretrovirus (δ, orange), epsilonretrovirus (ϵ, red), lentiretrovirus (Lenti, yellow), and spuma-like retrovirus (Spuma-like, purple). The final alignment length consisted of 240 taxa and 162 amino acids in length. The tree was constructed using RAxML 7.2.8-HPC2 on XSEDE [83]. The best substitution model was determined by ProtTest 2.4 [84] to be the LG + G + F model. The scale bar is in units of amino acid substitution per site. Numbers on nodes are bootstrap support values estimated using 5,000 pseudoreplicates. PSFVgal, PSFVaye, and ChrEFV are indicated in bold type and with asterisks. JSRV, jaagsiekte sheep retrovirus; MMTV, mouse mammary tumor virus; HERV, human endogenous retrovirus (ERV); LPDV, lymphoproliferative disease virus; EIAV, equine infectious anemia virus; BIV, bovine immunodeficiency virus; HIV, human immunodeficiency virus; SIV, simian immunodeficiency virus; BLV, bovine leukemia virus; HTLV, human T-lymphotropic virus; PFV, prototype FV; CoeEFV, coelacanth EFV; ZFERV, zebrafish ERV; SpeV, sphenodon ERV; MeEV, Meles endogenous virus (EV); VuEV, Vulpes EV; MLV, murine leukemia virus; MDEV, Mus dunni EV; MuRRS, murine retrovirus-related sequence; Xen, Xenopus laevis ERV; WDSV, walleye dermal sarcoma virus. (JPG 34 KB)

Additional file 4: Figure S3: Foamy virus (FV)-host evolutionary conflicts. Our analyses suggested two major FV-host evolutionary conflicts: one is of PSFVaye and RhiFV (**A**), and the other is of New World monkey (NWM) FVs (**B**). **A.1** shows our best estimated phylogeny in which both PSFVaye and RhiFV are placed robustly outside the Boreoeutherian FV clade but still remain together with exafroplacentalian FVs (see main text). **A.2-4** show alternative phylogenies in which either PSFVaye or RhiFV (**A.2 & A.3**) or both (**A.4**) co-speciate with their hosts. We used approximately unbiased (AU) [67] tests to compare these alternative phylogenies against one another given Pol-Env/Pol/Env alignments, based on comparisons of site-wise log-likelihood scores (LLH) computed using PAML 4.7a [87]. The AU tests were performed in Consel [88]. The same analyses were performed to compare our best estimates of the phylogenetic placement of NWM FVs which show FV-host evolutionary conflicts (**B.1**) against topologies that do not (**B.2**). Results are summarized in the table; only total LLH scores and AU probabilities (p-AUs) are shown. (JPG 75 KB)

Additional file 5: Figure S4: Inferred foamy virus (FV)-host co-speciation events using co-phylogeny reconstruction software Jane v4.0 [65]. The FV Pol-Env protein phylogeny (blue and green) was superimposed upon a previously published host phylogeny (black, published in [43]) and is scaled to host divergence times. The scale bar is in units of millions of years. Concatenated Pol-Env protein alignments (893 and 603 amino acids, respectively) were used to infer the FV trees. Solid circles represent FV-host co-speciation events, open circles represent host switching events, and an asterisk represents a lineage duplication event in the absence of host diversification which gave rise to two FV lineages sharing host species. The thick lines in the FV phylogeny correspond to the evolutionary period spanning about 30 Myr, during which the two lineages shared their host species. A hypothetical scenario of host switching of New World monkey FVs (as indicated by an orange ‘**?**’) is shown in an orange box. An alternative scenario is shown in Figure 4. (JPEG 43 kb) (JPG 44 KB)

Additional file 6: Figure S5: Co-speciation between lorisiforme foamy viruses (FVs) and their hosts. FV phylogeny (left) was estimated using an alignment of short integrase protein sequences of lorisiformes (indicated with asterisks, 85 amino acids (aa)) and concatenated polymerase-envelope protein sequences of other eutherian FVs (893 aa and 603 aa, respectively). The phylogeny was inferred using Bayesian methods in the program MrBayes 3.2.1 [85]) and rooted with CoeEFV. Numbers at branch nodes are posterior probabilities and the scale bar is in units of amino-acid substitutions per site. The topology of the lorisiforme FV phylogenetic relationships is compared to that of their prosimian hosts (right, previously published in [43]). Solid lines between the two trees indicate FV-host associations. (JPG 83 KB)

Additional file 7: Figure S6: Investigation of the effects of neutral genetic changes on foamy virus (FV) phylogenetic relationships. We mutated the polymerase and envelope nucleotide sequences of SFVagm, SFVsqu, and PSFVgal (indicated with asterisks) *in silico* with a substitution rate of 10E-9 substitutions per site per year over a hypothetical period of 40 Myr. The mutated gene sequences were then translated into protein sequences and the FV phylogeny was re-built as described using Bayesian methods using MrBayes 3.2.1 [85]. The substitution process was assumed to be neutral, homogenous, and independent across all sites and base types. Numbers at branch nodes are posterior probabilities and the scale bar is in units of amino acid substitutions per site. (JPG 25 KB)
