# Supplementary material for: Discovery of prosimian and afrotherian foamy viruses and potential cross species transmissions amidst stable and ancient mammalian co-evolution
Source: Retrovirology. 2014 Aug 4;11:61. doi: 10.1186/1742-4690-11-61 (PMC4261875; doi:10.1186/1742-4690-11-61)
Supplement: Supplementary file 1 — Additional file 1: Table S1: Mammalian foamy viruses. Table S2. Distribution of simian foamy virus in prosimians. Table S3. GenBank accession numbers of protein sequences used as probes to search for integrated mammalian foamy viruses (FVs) as well as for phylogenetic analyses. (DOCX 63 KB) [file 12977_2014_3914_MOESM1_ESM.docx]

**Table S1. Mammalian foamy viruses (FVs).**

| **FVs†‡** | **Host species** | **Host group** | **Year first isolate [ref]** | **Year first full-genome available [ref]** |
| --- | --- | --- | --- | --- |
| **Exogenous foamy virus** | | | | |
| SFVmac (SFV-1, SFV-2) | Macaque (*Macaca mulatta, M. cyclopis*) | Boreoeutheria | 1955 [1], 1961 [2] | 1991 [3] |
| SFVagm (SFV-3) | African green monkey (*Cercopithecus aethiops*) |  | 1964 [4] | 1992 [5] |
| SFVsqu (SFV-4) | Squirrel monkey (*Saimiri sciureus*) |  | 1971 [6] | 2010 [7] |
| PSFVgal (SFV-5) | Galago (*Otolemur crassicaudatus panganiensis*) |  | 1971 [6] | - |
| SFVcpz (SFV-6, SFV-7) | Chimpanzee (*Pan troglodytes verus*) |  | 1967 [8] | 1994 [9] |
| SFVspm (SFV-8) | Spider monkey (*Ateles sp.*) |  | 1973 [10] | 2007 [11] |
| SFVcap (SFV-9) | Capuchin (*Cebus sp.*) |  | 1975 [12] | - |
| SFVbab (SFV-10) | Baboon (*Papio cynocephalus*) |  | 1975 [13] | - |
| SFVora (SFV-11) | Orangutan (*Pongo pygmaeus*) |  | 1994 [14] | 2003 [15] |
| SFVgor | Gorilla (*Gorilla gorilla sp.*) |  | 1995 [16] | 2011 [17] |
| SFVmar | Marmoset (*Callithrix jacchus*) |  | 1981 [18] | 2010 [7] |
| FFV | Domestic cat (*Felis catus*) |  | 1969 [19] | 1997 [20] |
| BFV | Cow (*Bos taurus*) |  | 1969 [21] | 1994 [22] |
| EFV | Horse (*Equus ferus caballus)* |  | 2000 [23] | 2000 [23] |
| RhiFV | Bat (*Rhinolophus affinis)* |  | 2012 [24] | - |
| PFV* | Human (*Homo sapiens*) |  | 1971 [25] | 1987 [26], 1988 [27] |
| **Endogenous foamy virus** | | | | |
| PSFVaye | Aye-aye (*Daubentonia madagascariensis*) | Boreoeutheria | 2012 [28] | - |
| SloEFV | Two-toed sloth (*Choloepus hoffmanni*) | Xenarthran | 2009 [29] | 2009 [29] |

† Acronyms used in FV names: SFVmac, macaque FV; SFVagm, African green monkey FV; SFVsqu, squirrel FV; PSFVgal, galago prosimian FV; SFVcpz, chimpanzee FV; SFVspm, spider monkey FV; SFVcap, capuchin FV; SFVbab, baboon FV; SFVora, orangutan FV; SFVgor, gorilla FV; SFVmar, common marmoset FV; FFV, feline FV; BFV, bovine FV; EFV, equine FV; RhiFV, *Rhinolophus* FV; PFV, prototype FV; PSFVaye, aye-aye prosimian FV; SloEFV, sloth endogenous FV.

‡ Names within brackets are old nomenclatures based on serotyping which are not used anymore.

* Although the prototype FV (PFV) was isolated from a human and was originally called a human foamy virus (HFV), it is well established that it is in fact a variant foamy virus of chimpanzee origin, most closely related to and clustering well within the clade of foamy viruses from *Pan troglodytes schweinfurthii* [30–32]. Phylogenetic analyses revealed evidence for coevolution of chimpanzee hosts and their specific FVs at the subspecies level [30, 32]. Therefore, PFV is here treated as a *Pan troglodytes schweinfurthii* FV which diverged from SFVcpz about 0.96 million years ago, inferred under the FV-host co-speciation assumption using the divergence date of *Pan troglodytes schweinfurthii* and *Pan troglodytes verus* [33].

1. Rustigian R, Johnston P, Reihart H: **Infection of monkey kidney tissue cultures with virus-like agents.** *Proc Soc Exp Biol Med* 1955, **88**:8–16.

2. Johnston PB: **A second immunologic type of simian foamy virus: monkey throat infections and unmasking by both types.** *J Infect Dis* 1961, **109**:1–9.

3. Kupiec JJ, Kay A, Hayat M, Ravier R, Périès J, Galibert F: **Sequence analysis of the simian foamy virus type 1 genome.** *Gene* 1991, **101**:185–94.

4. Stiles GE, Bittle JL, Cabasso VJ: **Comparison of Simian Foamy Virus Strains including a New Serological Type**. *Nature* 1964, **201**:1350–1351.

5. Renne R, Friedl E, Schweizer M, Fleps U, Turek R, Neumann-Haefelin D: **Genomic organization and expression of simian foamy virus type 3 (SFV-3).** *Virology* 1992, **186**:597–608.

6. Johnston PB: **Taxonomic features of seven serotypes of simian and ape foamy viruses.** *Infect Immun* 1971, **3**:793–799.

7. Pacheco B, Finzi A, McGee-Estrada K, Sodroski J: **Species-specific inhibition of foamy viruses from South American monkeys by New World Monkey TRIM5{alpha} proteins.** *J Virol* 2010, **84**:4095–4099.

8. Rogers NG, Basnight M, Gibbs CJ, Gajdusek DC: **Latent Viruses in Chimpanzees with Experimental Kuru**. *Nature* 1967, **216**:446–449.

9. Herchenröder O, Renne R, Loncar D, Cobb EK, Murthy KK, Schneider J, Mergia A, Luciw PA: **Isolation, cloning, and sequencing of simian foamy viruses from chimpanzees (SFVcpz): high homology to human foamy virus (HFV).** *Virology* 1994, **201**:187–99.

10. Hooks JJ, Gibbs CJ, Chou S, Howk R, Lewis M, Gajdusek DC: **Isolation of a new simian foamy virus from a spider monkey brain culture.** *Infect Immun* 1973, **8**:804–813.

11. Thümer L, Rethwilm A, Holmes EC, Bodem J: **The complete nucleotide sequence of a New World simian foamy virus.** *Virology* 2007, **369**:191–197.

12. Hooks JJ, Gibbs CJ: **The foamy viruses.** *Bacteriol Rev* 1975, **39**:169–85.

13. Heberling RL, Kalter SS: **Isolation of foamy viruses from baboon (Papio cynocephalus) tissues.** *Am J Epidemiol* 1975, **102**:25–9.

14. McClure MO, Bieniasz PD, Schulz TF, Chrystie IL, Simpson G, Aguzzi A, Hoad JG, Cunningham A, Kirkwood J, Weiss RA: **Isolation of a new foamy retrovirus from orangutans.** *J Virol* 1994, **68**:7124–7130.

15. Verschoor EJ, Langenhuijzen S, van den Engel S, Niphuis H, Warren KS, Heeney JL: **Structural and evolutionary analysis of an orangutan foamy virus.** *J Virol* 2003, **77**:8584–8587.

16. Bieniasz PD, Rethwilm A, Pitman R, Daniel MD, Chrystie I, McClure MO: **A comparative study of higher primate foamy viruses, including a new virus from a gorilla.** *Virology* 1995, **207**:217–228.

17. Schulze A, Lemey P, Schubert J, McClure MO, Rethwilm A, Bodem J: **Complete nucleotide sequence and evolutionary analysis of a gorilla foamy virus.** *J Gen Virol* 2011, **92**:582–586.

18. Marczynska B, Jones CJ, Wolfe LG: **Syncytium-forming virus of common marmosets (Callithrix jacchus jacchus).** *Infect Immun* 1981, **31**:1261–1269.

19. Riggs JL, Oshirls, Taylor DO, Lennette EH: **Syncytium-forming agent isolated from domestic cats.** *Nature* 1969, **222**:1190–1.

20. Winkler I, Bodem J, Haas L, Zemba M, Delius H, Flower R, Flügel RM, Löchelt M: **Characterization of the genome of feline foamy virus and its proteins shows distinct features different from those of primate spumaviruses.** *J Virol* 1997, **71**:6727–6741.

21. Malmquist WA, Van der Maaten MJ, Boothe AD: **Isolation, immunodiffusion, immunofluorescence, and electron microscopy of a syncytial virus of lymphosarcomatous and apparently normal cattle.** *Cancer Res* 1969, **29**:188–200.

22. Renshaw RW, Casey JW: **Transcriptional mapping of the 3’ end of the bovine syncytial virus genome.** *J Virol* 1994, **68**:1021–1028.

23. Tobaly-Tapiero J, Bittoun P, Neves M, Guillemin MC, Lecellier CH, Puvion-Dutilleul F, Gicquel B, Zientara S, Giron ML, de Thé H, Saïb A: **Isolation and characterization of an equine foamy virus.** *J Virol* 2000, **74**:4064–4073.

24. Wu Z, Ren X, Yang L, Hu Y, Yang J, He G, Zhang J, Dong J, Sun L, Du J, Liu L, Xue Y, Wang J, Yang F, Zhang S, Jin Q: **Virome Analysis for Identification of Novel Mammalian Viruses in Bat Species from Chinese Provinces**. *J Virol* 2012, **86**:10999–11012.

25. Achong BG, Mansell PW, Epstein MA, Clifford P: **An unusual virus in cultures from a human nasopharyngeal carcinoma.** *J Natl Cancer Inst* 1971, **46**:299–307.

26. Flügel RM, Rethwilm A, Maurer B, Darai G: **Nucleotide sequence analysis of the env gene and its flanking regions of the human spumaretrovirus reveals two novel genes.** *EMBO J* 1987, **6**:2077–2084.

27. Maurer B, Bannert H, Darai G, Flügel RM: **Analysis of the primary structure of the long terminal repeat and the gag and pol genes of the human spumaretrovirus.** *J Virol* 1988, **62**:1590–1597.

28. Han G-Z, Worobey M: **An endogenous foamy virus in the aye-aye (Daubentonia madagascariensis).** *J Virol* 2012, **86**:7696–8.

29. Katzourakis A, Gifford RJ, Tristem M, Gilbert MTP, Pybus OG: **Macroevolution of complex retroviruses.** *Science* 2009, **325**:1512.

30. Switzer WM, Bhullar V, Shanmugam V, Cong M-E, Parekh B, Lerche NW, Yee JL, Ely JJ, Boneva R, Chapman LE, Folks TM, Heneine W: **Frequent simian foamy virus infection in persons occupationally exposed to nonhuman primates.** *J Virol* 2004, **78**:2780–9.

31. Meiering CD, Linial ML: **Historical perspective of foamy virus epidemiology and infection.** *Clin Microbiol Rev* 2001, **14**:165–76.

32. Liu W, Worobey M, Li Y, Keele BF, Bibollet-Ruche F, Guo Y, Goepfert PA, Santiago ML, Ndjango J-BN, Neel C, Clifford SL, Sanz C, Kamenya S, Wilson ML, Pusey AE, Gross-Camp N, Boesch C, Smith V, Zamma K, Huffman MA, Mitani JC, Watts DP, Peeters M, Shaw GM, Switzer WM, Sharp PM, Hahn BH: **Molecular ecology and natural history of simian foamy virus infection in wild-living chimpanzees.** *PLoS Pathog* 2008, **4**:e1000097.

33. Stone AC, Battistuzzi FU, Kubatko LS, Perry GH, Trudeau E, Lin H, Kumar S: **More reliable estimates of divergence times in Pan using complete mtDNA sequences and accounting for population structure.** *Philos Trans R Soc Lond B Biol Sci* 2010, **365**:3277–88.

**Table S2. Distribution of Simian Foamy Virus (SFV) in Prosimians.**

| **Common Name** | **Scientific Name** | **Origin** | **Serology^†^No. Pos/Total (%)** | **PSFVgal-specific PCRNo. Pos/Total (%)** | **PSFVaye-specific PCRNo. Pos/Total (%)** | **Generic NWM**‡ **SFV PCRNo. Pos/Total (%)** | **PSFVgal-PSFVaye-generic PCRNo. Pos/Total (%)** |
| --- | --- | --- | --- | --- | --- | --- | --- |
| Phillipine tarsier^1^ | *Tarsius syrichta* | wild | ND | ND | ND | 0/2 | 0/2 |
| Garnett’s great bush baby^1^ | *Otolemur garnetti* | captive | 11/14 (78.6) | ND | ND | ND | ND |
| Silvery greater galago^1^ | *Otolemur monteiri monteiri* | captive | ND | 0/5 | ND | 0/5 | 2/5 (40) |
| Silvery greater galago^1^ | *Otolemur monteiri argentatius* | captive | ND | ND | ND | 0/1 | 0/1 |
| Southern lesser bush baby^1^ | *Galago senegalensis moholi* | captive and wild | 5/7 (71.4) | 0/8 | ND | 0/9 | 4/9 (44) |
| Demidoff’s dwarf galago^1^ | *Galago demidoff* | captive | ND | ND | ND | 0/1 | 0/1 |
| Bosman’s potto^2^ | *Perodicticus potto* | wild | ND | ND | ND | 0/27 | 1/27 (3.7) |
| Aye-aye^1^ | *Daubentonia madagascariensis* | captive | 0/17 | ND | 18/18 (100) | 18/18 (100) | 18/18 (100) |
| Blue-eyed black lemur^3^ | *Eulemur macaco flavifrons* | captive | 0/4 | ND | ND | ND | ND |
| Mongoose lemur^3^ | *Eulemur mongoz* | captive | 0/5 | ND | ND | ND | ND |
| Red-collared brown lemur^1^ | *Eulemur fulvus collaris* | captive | ND | ND | ND | 0/1 | 0/1 |
| Red-bellied lemur^1^ | *Eulemur rubriventer* | wild | ND | ND | ND | 0/1 | 0/1 |
| Grey bamboo lemur | *Hapalemur griseus griseus* | captive | 0/6 | ND | ND | ND | ND |
| Ring-tailed lemur^1,3^ | *Lemur catta* | captive | 0/20 | ND | ND | 0/3 | 0/11 |
| Red-ruffed lemur^3^ | *Varecia variegata rubra* | captive | 0/32 | ND | ND | 0/20 | 0/20 |
| Grey-brown mouse lemur^1^ | *Microcebus griseorufus* | wild | ND | ND | ND | 0/3 | 0/3 |
| Grey mouse lemur^1^ | *Microcebus murinus* | captive | ND | ND | ND | 0/5 | 0/5 |
| Coquerel’s sifaka^1^ | *Propithecus verreauxi coquereli* | captive | ND | ND | ND | 0/1 | 0/1 |
| Diademed sifaka^1^ | *Propithecus tattersalli (diadema)* | captive | ND | ND | ND | 0/1 | 0/1 |

‘ND’= ‘not done’**;** when present in all three PCR result columns indicates only serum was available for testing. For pottos, only dried blood spots were available.

†Western blot testing using antigens from an PSFVgal-infected cell line.

‡NWM, New World monkey

^1^Samples from Duke Lemur Center, ^2^Cameroon, ^3^various U.S. zoos

**Table S3. GenBank accession numbers of** **protein sequences** **used as probes to search for integrated mammalian foamy viruses (FVs) as well as for phylogenetic analyses.**

| **FV**† **(Accession number)** | **Gag** | **Pol** | **Env** | **Bel-1** | **Bel-2** |
| --- | --- | --- | --- | --- | --- |
| PFV (Y07725) | CAA69002 | CAA69003 | CAA69004 | CAA69005 | NA‡ |
| SFVcpz (U04327) | AAA19977 | AAA19978 | AAA19979 | AAA19980 | AAA19981 |
| SFVgor (HM245790) | ADN65590 | ADN65591 | ADN65592 | ADN65594 | ADN65593 |
| SFVora (AJ544579) | CAD67561 | CAD67562 | CAD67563 | CAD67564 | CAD67565 |
| SFVagm (M74895) | AAA47795 | AAA47796 | AAA47798 | AAA47799 | AAA47800 |
| SFVmac (NC_010819) | YP_001961121 | YP_001961122 | YP_001961123 | YP_001961124 | NA |
| SFVmar (GU356395) | ADE05999 | ADE06000 | ADE06001 | ADE06002 | ADE06003 |
| SFVsqu (GU356394) | ADE05994 | ADE05995 | ADE05996 | ADE05997 | ADE05998 |
| SFVspm (EU010385) | ABV59398 | ABV59399 | ABV59400 | ABV59401 | ABV59402 |
| BFV (U94514) | AAB68769 | AAB68770 | AAB68771 | AAB68772 | AAB68773 |
| EFV (AF201902) | AAF64413 | AAF64414 | AAF64415 | AAF64416 | AAF64417 |
| FFV (Y08851) | CAA70074 | CAA70075 | CAA70076 | CAA70077 | CAA70078 |

‘NA’ = ’Not available’.

†Acronyms used in FV names: PFV, prototype FV; SFVcpz, chimpanzee FV; SFVgor, gorilla FV; SFVora, orangutan FV; SFVagm, African green monkey FV; SFVmac, macaque FV; SFVmar, common marmoset FV; SFVsqu, squirrel FV; SFVspm, spider monkey FV; BFV, bovine FV; EFV, equine FV; FFV, feline FV.
