# Supplementary material for: Discovery of prosimian and afrotherian foamy viruses and potential cross species transmissions amidst stable and ancient mammalian co-evolution
Source: Retrovirology. 2014 Aug 4;11:61. doi: 10.1186/1742-4690-11-61 (PMC4261875; doi:10.1186/1742-4690-11-61)
Supplement: Supplementary file 2 — Additional file 2: Figure S1: Detailed descriptions of PSFVgal, PSFVaye, and ChrEFV complete or partial genomes. Protein open reading frame (ORF) and putative reading frame (RF) locations were determined by sequence similarity to other FVs and by searching against the GyDB (http://www.gydb.org/). Short interspersed nuclear elements (SINEs) were determined by CENSOR (http://www.girinst.org/censor/). TATA boxes, polyadenylation signals, primer binding sites (PBS), and polypurine tracts (PPT) are indicated by bold type and solid lines. Long terminal repeats (LTRs), Gag, Pol, Env, Bel1, and Bel2 proteins are highlighted in grey, green, purple, blue, orange, and red, respectively. Several conserved domains and catalytic centers are highlighted in darker colors. Putative insertion and deletion mutations are indicated by red dashes. The beginning and the end of ORFs and RFs are indicated by arrows and square-arrows, respectively. (PDF 386 KB) [file 12977_2014_3914_MOESM2_ESM.pdf]

PSFVgal

5′-LTR

1 **TG**TCATGGGAAAAAGGTCTCTTGACTAGGAATTTTAGCTACTTTAGATCTAGTGCTGTGAGACCACAGGAATTGGAAGCT 80

81 ATGGTAAATTCCAAATTGGTATTGGCCCCTCTTCATCCCTTCCAGTACAGAGCTGGTATACAGCTCTGGGTTAATACAGC 160

161 CTTGCCCCCCGACTGGGTTGTACTGGATCCCAGAGGAAATAGGTGGGTGTCTTCAGGACAAGGCTGGGAACAAGTGTATG 240

241 AGGACATAGTGGACTGTGCCGACCCTGAACACTTCACCTCAGGTGATCCAGGTATGACATTGCCTCTGCATATCATACCG 320

321 GAGGAATGTAGGGATGATTATGAGAGTGACGGGGAAATACCACGTCACCGAGGCCTTGGCTGGGTATCAGATCCTGAAAG 400

401 CCCCAGGCCTCCTAGTCCCTCCAACAGCACTATGTCGGCTTCGCCACTAGATGGAGCCTGCTCCGAAAAAGAAGACTGGG 480

481 AGGAACCACCTCCTCTCAGACACAGGCCTTCCACTCCTTATCCACCTCCTCCTCCTGAAAGTAATGAAAGTGAGGAGGAT 560

561 TAAGGGATTAGTGCTGTAGCATATTGGCACAGTCCTAGTTGAGGGTTACGCAGCCCACTTGACTCTGGCAGTTCGAATGA 640

641 CTCAAGTGGATGTGTAGCCTTTTGCCAGCAATTGCAGTATTACCACATATAGCTTTGCTACATATTGTTATTTGCCATAG 720

721 TACTTAAGGGATTAATGGTCTAGCAAGATCACTAGGAGCTAGGCTAGCAATCCAGTTAGACCATTAGTCACTTGACATTC 800

801 ACACTGCAAATGTGAGTTCGAGTCTTTGTAGTGTGGGGTGTTGAGTTTACCAGCTTTTGCTAATGGAAATCCATACATGG 880

TATA box **U3/R boundary**

881 TATTGGTACCGATAATTGTATTTCCAATGATTGTATTAATGTTTTAG**TATATAA**GGATCCCAAATAGATTGTACGGGCCC 960

961 CCACTCACTTCAGCTGGCTGCGACCAGTGTGAGATTGGTGTCTCCAGACTTGGTAAGAGATTTTTATCATTATATTGTAA 1040

Polyadenylation signal **R/U5 boundary**

1041 GGCTTGGATAATTAATTGTTAGATGCTCTGCTATTAAT**AATAAA**TTGGTTGTATTTTGAGAACCACAATTTAACTATTGT 1120

1121 CTCTCTTTATTCCTCCTGGTTGAGGACAAGGGAACCTCTCCCTCTCGGGTCTTGCAGTAATAAATAAGTATAAGGGGGAG 1200

PBS

1201 ACTAAGTCTCTGACATATTATAAAAATACTTATTTATAAGATCCTTTGAGAGGGTGAGTCCACGA**CA**AT**TGGCGTCCCTG** 1280

1281 **GGTGGGC**TCGAAATACAACCAAAAGTGCCTTATTGTTGTGAGTCATAAGGTTATACCCTAGGGGTCTTTGGCACAGTCAA 1360

Gag ORF

1361 AGATGTCTCAGCCCTCGGCTTCCGGTTCAGCTGGCGCTGGTGGAGCTCCTCAGCAGCCCCCTCCACCGCCACCTCAGCCC 1440

M S Q P S A S G S A G A G G A P Q Q P P P P P P Q P

1441 GGCCCAGCGGCTCCAGTGCCACGAGCACAAATTGGTTATGGTGACCTGGACGTACTTTTATTACAACAGGAATATCATTT 1520

G P A A P V P R A Q I G Y G D L D V L L L Q Q E Y H L

1521 GATAGATCCAAATTTACAGGTACAACACTTAGACACTTTATTAGTTAGAATCACAGGAGGCAACTGGGGACCAGGTGACC 1600

Cytoplasmic

I D P N L Q V Q H L D T L L V R I T G G N W G P G D R

1601 GATTTGCTCGTATTGAGGTTCTTCTTAGGGACACCCTAGGACCACTCCAACAACCCAGGTATAGGTATGCAGCAATGCAG 1680

retention signal

F A R I E V L L R D T L G P L Q Q P R Y R Y A A M Q

1681 CAAGCAGATCTCAGGAATGACATCATCCTGCATCTAAATTATCAAGATGCAATTATAATTTTTGATATGATAATACCATC 1760

Q A D L R N D I I L H L N Y Q D A I I I F D M I I P S

1761 TGAAGGAGTTCATAGGCATGGACCCATGTTCGATGGACTTTGGATTCATGGAGATGATTACTCCATGAACTTTCAGCCAA 1840

E G V H R H G P M F D G L W I H G D D Y S M N F Q P I

1841 TTACAGCACATGAACTGTATCTTTTGCCACAGCAAGTTCTCACAGAAGAAGTGGAACTGCTGACGGAAGTGTGCAACCGA 1920

T A H E L Y L L P Q Q V L T E E V E L L T E V C N R

1921 ATGGCAGACTGGATAAGACGGCATCGCTGTGGAGGAGGAAGTGGAAGCTCACAACCCCCGCCGCCGCCTCCACCCGCTGT 2000

M A D W I R R H R C G G G S G S S Q P P P P P P P A V

2001 GCCTGTCTTGCCTTCAGCACCTCCGGCTAGCAGTTTACCCTTACCACCACAAGGATGGGGAATCTCCCCACCAGTAGCCA 2080

PSAP motif

P V L P S A P P A S S L P L P P Q G W G I S P P V A T

2081 CATCCACTCCAGGAGCGGCAGGACATAGCTCCAGTGCCGGCCCTAATATTTCCCTTGGTGGAACATATGTTCCTCCACCG 2160

S T P G A A G H S S S A G P N I S L G G T Y V P P P

2161 GTAGCACCACCAGCTCCGGTGATAGGGGGACCCGGAGGTCCCGGACAGTTGCCAGCAATGGTGCAAGTCCTCCCCGCTCA 2240

V A P P A P V I G G P G G P G Q L P A M V Q V L P A Q

2241 GCCAGTAGTAATTCCCATCAATGTTATCCGAAGTGTTTGTGGAGATACTCCATCAAACCCACAGGATATTCCATTGTGGA 2320

P V V I P I N V I R S V C G D T P S N P Q D I P L W M

2321 TGGGAAGAATTATTCCAGCCATTGAGGGTGTGTTTCCTATTGATAATCCAAATCTACGAATGAGAGTTGTGAATGCCCTG 2400

G R I I P A I E G V F P I D N P N L R M R V V N A L

2401 CTTGCCCTACATCCGGGCTTAGCAATAACAGAACTTAATGCCCAGACTTGGGGACAAGTCTTGGCTGTGCTCCATATGCG 2480

L A L H P G L A I T E L N A Q T W G Q V L A V L H M R

2481 AGCCTTGGGACACACAGCCCTCCATCAGCTTCCTGCACTCTTGGAGACTATTGTCAAGACAGATGGCATATTGCCAGCAT 2560

A L G H T A L H Q L P A L L E T I V K T D G I L P A Y

2561 ATAATATGGGTATGGAAGTAACTCAACAAGACTTTTCATACGTATGGGGAATTTTAAGAACACTACTTCCGGGACAAGCA 2640

N M G M E V T Q Q D F S Y V W G I L R T L L P G Q A

2641 TTTGTCCTAAGTATGCAAAATGAATTAGATAGATTACCTGCGGCTCAGAGACCGGGAATGTTTCCCGGCCTACTGCAGAG 2720

F V L S M Q N E L D R L P A A Q R P G M F P G L L Q R

2721 GACTCTGGACATACTAGGGTTAAATTCTCGGGGACAAAATATTCAAAAAACCAATACTCAGCAACAAGCTCCAAAAAGGG 2800

T L D I L G L N S R G Q N I Q K T N T Q Q Q A P K R G

2801 GTCAGAAACCGAAACCTCGACTTCCTCCTGTGCATAGGCGACCCGCACCATTTACACCACCTGCTACCCCTAGCCCGCGA 2880

Glycine-arginine box I

Q K P K P R L P P V H R R P A P F T P P A T P S P R

2881 CAACAAGCTTCTGCTTCCCCTTCTTCGCAGGGCGATAACCGAAGTCCTCAACCCCAAGGGCGTGGTACTTACGGACCGTC 2960

Q Q A S A S P S S Q G D N R S P Q P Q G R G T Y G P S

2961 CAGAGGAGGAGGCAGTGGGCCCAGATATAACTTCAGGCCCCGAGTGCAACCACCTGATCGATACGGCTTTGGAAGGGGAC 3040

Glycine-arginine box II

R G G G S G P R Y N F R P R V Q P P D R Y G F G R G Q

3041 AAGGGGGTCGCAGTTCAATAGGTGCTCAAGATAATCAACAGCCTGGTCAAGGAGGACAACGCACTCAACAAACAAACCAA 3120

Glycine-arginine box III

G G R S S I G A Q D N Q Q P G Q G G Q R T Q Q T N Q

3121 AACCGAAACCAAGGCAACGCCACGGGAGGAAGAACTCAACCTCAAAATCGCACTGTGAATACTGTGCGTGTCACACAGAC 3200

N R N Q G N A T G G R T Q P Q N R T V N T V R V T Q T

Pol ORF (uncertain starting site)

3201 TAACCCCCAAGGAGGAAGTTCAGTGTCAAACCCAGCTGTGACAACTTCTCAGAATACAGGAACAGGAAGTGCAACTCAGA 3280

N P Q G G S S V S N P A V T T S Q N T G T G S A T Q S

L R I Q E Q E V Q L R

Gag ORF end

3281 GCTCATCATCTTAAAGGCTTTGTGGACACAGGAAGCTCGATAACTTGTTTTCCAAAGTATACATTGGTAGAAGAAGATCC 3360

S S S *

Protease catalytic centre

A H H L K G F V D T G S S I T C F P K Y T L V E E D P

3361 AATTGGACAATATGATATCTCCACTATCCATGGAACAGTCTCACAGCCTGTATATTATATTAAGTTTAAAGTTAATGGTA 3440

I G Q Y D I S T I H G T V S Q P V Y Y I K F K V N G K

3441 AAAAGGTAGAAGCTGAAGTTACTGAGTCACCTTTGGACTATGTGATTTTATGCCCAAGTGATGTTCCGTGGCTCAGCACG 3520

K V E A E V T E S P L D Y V I L C P S D V P W L S T

3521 AAATTAGAATTAACTATTAAATTACCGCTCCACCAACAGCAGCAGGAATTATTAAGCAAAACAAGAGTAAATCAAAAACA 3600

K L E L T I K L P L H Q Q Q Q E L L S K T R V N Q K Q

3601 ATTACAGAAATTGTTTATACAATATGATGATCTCTGGCAGAAGTGGGAAAATCAAGTCGGCCATAGGAAAATAACCCCAC 3680

L Q K L F I Q Y D D L W Q K W E N Q V G H R K I T P H

3681 ATCATATTGCAACAGAAACTGTAGCTCCAAAACCACAAAGGCAATACCATGTCAACCCAGCTGCAAGGCCCGATATTCAG 3760

H I A T E T V A P K P Q R Q Y H V N P A A R P D I Q

3761 ATTGTTATAGATGACTTATTAAAGCAAGGCGTTTTGAAAAAGCAAAACAGCCCGATGAACACTCCGGTGTATCCGGTACC 3840

I V I D D L L K Q G V L K K Q N S P M N T P V Y P V P

3841 TAAACCTGATGGTAAATGGAGAATGGTCCTAGATTATAGAGCTGTTAATAAGACAGTTCCAGCTATAGGTGCGCAAAATT 3920

K P D G K W R M V L D Y R A V N K T V P A I G A Q N C

3921 GTCACGCACCCGGTATATTAAGCTCATTATACAGAGCTAAATTCAAAACAACTTTAGATTTAAGCAATGGGTTCTGGTCA 4000

H A P G I L S S L Y R A K F K T T L D L S N G F W S

4001 CATCCCATAACTCCTGAGTCATACTGGCTTACCGCCTTCACGTGGCAAGGCTCCCAGTATGTCTGGACTAGGTTACCCCA 4080

H P I T P E S Y W L T A F T W Q G S Q Y V W T R L P Q

4081 GGGATTTTTAAATAGCCCAGCCTTATTCACGGCAGATGTAGTTGATCTATGTAAACACATACCTAATGTCTCTGCCTACG 4160

Reverse transcriptase

G F L N S P A L F T A D V V D L C K H I P N V S A Y V

4161 TGGATGATATCTACGTAAGCAATGACACAGCAGAAGAACATCTACGTACATTAGAACAGCTGTTTCGGACACTAATGTCT 4240

catalytic centre

D D I Y V S N D T A E E H L R T L E Q L F R T L M S

4241 GCAGGGTATATTGTGTCTTTAAAAAAGAGTAAAATTGGAGTCTCAGCTGTGGATTTCTTGGGTTTCGAAATCACTGATGA 4320

A G Y I V S L K K S K I G V S A V D F L G F E I T D D

4321 TGGCCGCGGCCTAACTTCCGCATTTAAAGAAAAATTAGTAAATATACAACCCCCCAGCTCGTTGAAACAACTACAAAGCA 4400

G R G L T S A F K E K L V N I Q P P S S L K Q L Q S I

4401 TCTTGGGATTTCTAAACTTTACAAGAAATTTTGTACCAAATTATTCTGAGCTGGTCAAGCCACTGTACAATCTCGTTGCT 4480

L G F L N F T R N F V P N Y S E L V K P L Y N L V A

4481 ACTGCTCAAGGAAATAGAATCTCTTGGACTTTAGAAAATACAGAACAATTAAACCAAGTTATTTCGGCTTTAAACGCAGC 4560

T A Q G N R I S W T L E N T E Q L N Q V I S A L N A A

4561 AGATAACTTATCAGAAAGAAAGACAGGCGTCCCCTTGGTGGTTAAAAGCAATTGTTCCCCTACGGCGGGGTACATTCGGT 4640

D N L S E R K T G V P L V V K S N C S P T A G Y I R F

4641 TCTACAATCAAGGAGACCGAAAACCTATTCAATATGTAAATTATATTTTTTCAAAAACTGAATTGAAATTTACTCCTCTA 4720

Y N Q G D R K P I Q Y V N Y I F S K T E L K F T P L

4721 GAAAAACAATTGACAGTATTGCATAAGGCAATCCTAAAAGGATTAGATTTAGCTGGAGGAGAAGATATACATTTTTATAC 4800

E K Q L T V L H K A I L K G L D L A G G E D I H F Y T

4801 ACCAATTGCTTCTATTTCCAAGCTACAGAGAACTCCAATTCCAGAAAGGAAAGCGCTCCATGTCCGATGGCTTACATGGA 4880

P I A S I S K L Q R T P I P E R K A L H V R W L T W I

4881 TCACTTATTTAGAGGACCCGCGGTTTCATTTTTATTATGATGAAACCTTACCACCATTAGCTGAGCTCCCTGAAATTGCT 4960

T Y L E D P R F H F Y Y D E T L P P L A E L P E I A

4961 CAGGGTAACCAGCTATTACCATTAAAAGAATATACATCTGTGTTTTACACAGATGGGTCAGCAATAAAAAATCCTAATCC 5040

RNase H active site

Q G N Q L L P L K E Y T S V F Y T D G S A I K N P N P

5041 AAAGAAGGCACATTCTGCAGGTATGGGCACAGTAGAAGTCACCTACAATCCTGAATATAAGGTCCTCCATGAATGGAGCT 5120

K K A H S A G M G T V E V T Y N P E Y K V L H E W S F

5121 TCCCATTGGGTGATCACACAGCACAATATGCAGAAATCGCCGCGTGTGAATTTGCAATAAAAAAGGCCTCTCTTTTGCGA 5200

P L G D H T A Q Y A E I A A C E F A I K K A S L L R

5201 GGCCCAGTGCTGATAGTTTCAGATAGTGTTTACCTGGTTAAATCCTTTAATGAAGAGTTGCCTTTTTGGATCTCTAATGG 5280

RNase H active site

G P V L I V S D S V Y L V K S F N E E L P F W I S N G

5281 TTTTCTAAATAATAAAAAGAAACCATTGCAACACATAAGTAAATGGAAAACTATAGCTGCTTGCTATCAGAATAAGAAAG 5360

F L N N K K K P L Q H I S K W K T I A A C Y Q N K K D

5361 ATATATTTTTATTACATGTTCCAGGTCATCAGAAATTACTCACAGATGAACATGCCCAGGGGAATGCACTTGCAGATAAA 5440

I F L L H V P G H Q K L L T D E H A Q G N A L A D K

5441 CTTGCTGTGCAGAGCAGTCACAAGGTGTTGTTTATTAAAAAACCAAGTCTGGATGCAGAGCTTATCCAAGTCATGGAGGG 5520

L A V Q S S H K V L F I K K P S L D A E L I Q V M E G

5521 AAAATACCCTAAAGGGTACCCTCATAAATATGTATATGCACAAGATAATGGCAAAATCATTGTTACCTTGCCTAATGGTT 5600

K Y P K G Y P H K Y V Y A Q D N G K I I V T L P N G L

5601 TACAACGAGAGATACCCCCTGTAGGTGACCGACTGGCCCTTATTACTAAGGCCCATAACATTTCTCATATGGGCCGAGAG 5680

Q R E I P P V G D R L A L I T K A H N I S H M G R E

5681 GCAGTCTTGGCAAAAATTCAAAATGTATACTGGTGGCCTAATATGAAAAAGGATGTGAAACATGTTCTCACAATCTGTTC 5760

A V L A K I Q N V Y W W P N M K K D V K H V L T I C S

5761 TCAGTGTCAGCAAGTGAATTCCTTTAATTTAAAGCCACAGCCACCTCAAACTATTGCCAGACATGTACATCCTTTTGATA 5840

Q C Q Q V N S F N L K P Q P P Q T I A R H V H P F D K

5841 AAATCTATATGGATTATATTGGGCCTTTGCCACCCTCAGATGGGTATCTCTACGTTCTAGTGCTTGTTGACTCATGTACA 5920

I Y M D Y I G P L P P S D G Y L Y V L V L V D S C T

5921 GGGTTTACATGGCTCTACCCCACCAAGGCACCTTCTGCCAATGCAACTGTAAAAGCTCTCACTCATTTGACTGGTACTGC 6000

G F T W L Y P T K A P S A N A T V K A L T H L T G T A

PPT

6001 TGTTCCAAAGGTGCTGCATTCTGATCAAGGATCAGCATTCACCTCTTCCACCTTGGTTGATTGGGCC**AAGGAGAGGG**GCA 6080

Integrase active site **Integrase active site**

V P K V L H S D Q G S A F T S S T L V D W A K E R G I

6081 TACGTTTGGAATACAGTACTCCGTATCACCCCCAAAGTAGTGGAAAGGTGGAACGGAAAAACAGTGAAATAAAACGACTT 6160

Integrase active site

R L E Y S T P Y H P Q S S G K V E R K N S E I K R L

6161 TTAACTAAACTATTGGTTGGAAGACCCTTAAGGTGGTATCCATTAATCCCTACTGTTCAACTAGCACTTAATAACACGCC 6240

L T K L L V G R P L R W Y P L I P T V Q L A L N N T P

6241 AAATGTATCTTCAGGTAAAACCCCTCATCAATTGTTATTCGGGGTTGATTGTAACCTTCCTTTTGCTAATAAGGATACCT 6320

N V S S G K T P H Q L L F G V D C N L P F A N K D T L

6321 TAGATTTAACTCGAGAAAAACAGTTATCTCTCCTCAAAGAGCTTAGAGAAGATTTGGCTGCTTTGCCTTCCTCCTCCCCA 6400

D L T R E K Q L S L L K E L R E D L A A L P S S S P

6401 CCTCCACCTTCTCGTTCCTGGCGTCCTTCTGTTCGCCTGCTTGTCCAGGAGAGGGTCTATAGGCCCTCGGCCCTGAGGCC 6480

P P P S R S W R P S V R L L V Q E R V Y R P S A L R P

6481 CAAGTGGAGAAAACCAACACCTATTCTTGAAGTACATTCAGACCGCCTGGTAACAATCAAAGATCACCTTGGTAATATCA 6560

K W R K P T P I L E V H S D R L V T I K D H L G N I K

Env ORF

6561 AAAAGGTCAGTACCGATAATCTGAAACTAACCCCACATCAGGATGGAGGAACCAATGACACTCCAGCAGTGGTTAAGATG 6640

K V S T D N L K L T P H Q D G G T N D T P A V V K M

WXXW motif

M E E P M T L Q Q W L R W

Pol ORF end

6641 GAGAAACATGAACCTGTTGAACAAAGCAGACCAACTACAGAGGAAAGTACAACAAACACCCACGCTGACCAAAAGTGAAA 6720

E K H E P V E Q S R P T T E E S T T N T H A D Q K *

R N M N L L N K A D Q L Q R K V Q Q T P T L T K S E T

6721 CCCTGGAAGAAATCGTGGACATAGAACCACCGGTATATGACTCTACAACTGAGCCTTTACCCTATACAAGGATGGACCTA 6800

L E E I V D I E P P V Y D S T T E P L P Y T R M D L

6801 ATCAGGTTTCGCCTGTATAGATTATGTGCAACCACAACCCGGACAATGGGATGGTGCATAGGGTTGTTTTGTTTATTATT 6880

Hydrophobic transmembrane region with

I R F R L Y R L C A T T T R T M G W C I G L F C L L L

6881 GATTTTATTATTCTCCCTTGTGATTGTGATACTCAGGTTACAATGGCGCAATGCTATTGTTACCCCTGGGCCGATCATTG 6960

a signal-peptide peptidase cleavage site

I L L F S | L V I V I L R L Q W R N A I V T P G P I I A

6961 CCTGGAATGAGTCGCATGAAGCTGTCCTAACCACAAGGCCTAACTATACTGCTGCCAGGTCTCGACGTAGTGTGGAGCCT 7040

Furin-cleavage site

W N E S H E A V L T T R P N Y T A A R S R R | S V E P

7041 GCATTACCTGTGGACATAGAAATTAACATAACGGCCCTCCCTCAAGGAATGCTGCTGGTCCCACATACAAAACCTGTAGT 7120

A L P V D I E I N I T A L P Q G M L L V P H T K P V V

7121 AAAGAAGGAGAGGGCCTTAGGGTTCTCCCAAATCATAATTATGAGTTCTGATAGTATGGCCAATAGTATGGGCCTAAAGA 7200

K K E R A L G F S Q I I I M S S D S M A N S M G L K K

7201 AAGAAGACATACATCTCCTAGTGGACCTCCTCAATGAGGAAATGGAACAGTTACAAAATATTATATTAGAGTTTGATCTC 7280

E D I H L L V D L L N E E M E Q L Q N I I L E F D L

7281 CCTATTGGGGACCCTCATGATCAAAGTTATTATATAGAGCAACGCTGTAAGGCAGCCCTCCAACATTGCTATGTGGTGGA 7360

P I G D P H D Q S Y Y I E Q R C K A A L Q H C Y V V E

7361 AAGGGAAGGCAAGGGATGGCCCACAGATGGCGCCATCTTAGATCAATGCCCTCTTCCAGACATCACAAAGTGGAATCCAT 7440

R E G K G W P T D G A I L D Q C P L P D I T K W N P Y

7441 ATCAGACCCATGCCATATGGGACTACTATCTACAACCACCTGCCTTCGTAAAAAAGACATGGAACAGTTCCCAGGTATAT 7520

Q T H A I W D Y Y L Q P P A F V K K T W N S S Q V Y

7521 GGCAATGTCCGGATGGGTTCCTTCTACACAAAGGGTAATGGATTCCAAAATACTTCACAATATGTAATCTTTTGCTCTGA 7600

G N V R M G S F Y T K G N G F Q N T S Q Y V I F C S D

7601 CCAATTATATGGCTCATGGTATTATAAAAATGATGTAACACAACAAATGAGGGATCAGACGCTCATGTTAAAATTAAGAA 7680

Q L Y G S W Y Y K N D V T Q Q M R D Q T L M L K L R N

7681 ATCTTACTATGAGTAACACATCACATGCGCAGTTAAAAGACCGCGCCTTACCACCTGACTGGACAACTCAAGGCCAAAAT 7760

L T M S N T S H A Q L K D R A L P P D W T T Q G Q N

7761 CGGTTGTTTAGAAGCATAACAACATTTGATGTCTGCCAGCGACCGGAAATGGTCTTTCTGCTTAATACTACATATTATAC 7840

R L F R S I T T F D V C Q R P E M V F L L N T T Y Y T

7841 TTACTCATTATGGGAAGGAGATTGTAATATTACAAGCACTAATGTTTCTCTCCATCCCAGCTGCAAACATTTCAATAAAA 7920

Y S L W E G D C N I T S T N V S L H P S C K H F N K S

7921 GCACCAATAGACATCCATATGCATGTAGGCATTGGGGATTGTATTTTGGAGAGGAGAAGACTTTATGCTATTCAGATAGT 8000

T N R H P Y A C R H W G L Y F G E E K T L C Y S D S

8001 GAAGACAGATGTTCATACTTCCCATCATATTATGGAAGGGAGGCTCTGAACGATTTTGGATACCTCGCCTTCACAGACAT 8080

E D R C S Y F P S Y Y G R E A L N D F G Y L A F T D M

8081 GTTTCCGGCTCCAACCTGTATTGAGACAAAGGAAGTACGTAAACCACAATATAAGGTATATTCGGCCTTCCAAGAATGCA 8160

F P A P T C I E T K E V R K P Q Y K V Y S A F Q E C M

8161 TGATTAAAAGTCAACAATATGACATCAATGATGTCATAGCAAAATTGGAGGCCTTATTCACCCCTTTACAGGGGAGGCCT 8240

I K S Q Q Y D I N D V I A K L E A L F T P L Q G R P

8241 CAAAATAGAGCCTTTATGATAAGTTCCACTGGTATGCCTGAGGAGTATAAGGAGAAACCCATAAAAGTGCAAAGGTCCTG 8320

Q N R A F M I S S T G M P E E Y K E K P I K V Q R S C

8321 CCTAACATCAAGGAAGCGACGAGATACTAATTTCCATAAAATACAAAGCATAGGATTTAATTTAGCCAATGCCATTAGTA 8400

L T S R K R R D T N F H K I Q S I G F N L A N A I S T

8401 CTGTCTCAAAGATATCCGACTTGAATGATAATCAATTGGCAAAAGGCATGCATGTGCTACGAAATCATCTTGTCACCTTA 8480

V S K I S D L N D N Q L A K G M H V L R N H L V T L

8481 ATGGAAGCCACATTACATGATATCAGCAAATTTGAGAGTGGGCTTGCTCTACAACATTTGCATACGCACCTGGCACAATT 8560

M E A T L H D I S K F E S G L A L Q H L H T H L A Q L

8561 GCGAAGCACCCTCCAGGAAAACAGAGTGGATTGGAGTATACTGGATACAGCCTGGATTCAATCAGAATTAAATACTGATG 8640

R S T L Q E N R V D W S I L D T A W I Q S E L N T D D

8641 ACAATACCATGAAATTAATTAAGAGAACTGCAAAGGCAATGGTGCATCATGTCCAGCAAACCCAGAAGAGTTTAAGAGCC 8720

N T M K L I K R T A K A M V H H V Q Q T Q K S L R A

8721 ACAACCCGAGAAGTAGGGATCTACTTTGAAATTATCATTCCAGCAGCCATTTATACCCAGAATTGGCAGCCCTTAAATTT 8800

T T R E V G I Y F E I I I P A A I Y T Q N W Q P L N L

8801 AGGCCACTTGGTGTTCAACTCAGGACAGTTGACCCAAGTGTTTGTGGAACAACCTTACAACCTGGTAAGCATGGAATGTA 8880

G H L V F N S G Q L T Q V F V E Q P Y N L V S M E C N

8881 ACATTCCAACCTATTTACATATTGAGGAATGTGTCAATCAAGACTATCTCATATGTGACATTGTGGAGGAAGTACTTCCA 8960

I P T Y L H I E E C V N Q D Y L I C D I V E E V L P

8961 TGCGGAAATCAAACAGGAAGTGACTGTCCAGTAATGGCTAAGGCTGTTAAAGCCCCTTTTGTATCCATAACACCCCTTAA 9040

C G N Q T G S D C P V M A K A V K A P F V S I T P L K

9041 GAATGGGTCTTATGTGATTTTAGCAGACACATCAGCCTGCACAATACCAGCATATTCGCCAGTACTGGTAACCACAAATG 9120

N G S Y V I L A D T S A C T I P A Y S P V L V T T N D

9121 ACACGTTGCAATGTTATGGTCACATACTTAAGAGACCTTTAAAAAACCACAAAGATTTACCAGCTGTAACTATTTATGAA 9200

T L Q C Y G H I L K R P L K N H K D L P A V T I Y E

9201 CCACGAGTTCCAGACCTTGTAATCCGGCTGCCACATTTGGTAGGAGTGATAGCTCAGCTAAAAGATCTCAAGTTTCAAGT 9280

P R V P D L V I R L P H L V G V I A Q L K D L K F Q V

Internal promoter

9281 AACATCCTCTTGGGAGAG**TATAAAA**GACCAAATTGCAAGAAGCAAAGAGCTACTCTTACAGCTAGACCTCCACGAAGGAT 9360

T S S W E S I K D Q I A R S K E L L L Q L D L H E G S

9361 CAGCTCCAGAGTGGATCAACAGACTTGCTGCTGCAGCCGCTGACATTTGGCCAGCTACTGGACAAGCTCTTAAAGGACTA 9440

A P E W I N R L A A A A A D I W P A T G Q A L K G L

9441 GGAGACTTCTTGCAGAGTACGGTAGGGTCCCTGTTAGGGACTGGGCTTTCCTTCCTCTCCTACCTAAAACCAATCCTAAT 9520

G D F L Q S T V G S L L G T G L S F L S Y L K P I L I

Bel1 ORF

9521 AGGAATAGGACTTATATTCCTTGTTGTCATCCTATTTAAGATAATATCATGGCTTCCTGGAACCCGGGACAAAAGGGAAT 9600

G I G L I F L V V I L F K I I S W L P G T R D K R E *

M A S W N P G Q K G I

Env ORF end

9601 AAATATTCCCCAGAATGATGAGTCAGATCTGCCACAGCCAAGACCGCTGCCAGCACTGCCTGCTTCAGAATTCATGGACC 9680

N I P Q N D E S D L P Q P R P L P A L P A S E F M D H

9681 ATAATTATGTGGGAATGAGACAATGGTACAACGATCCCTCTGGGGGATATCCTGATTTAGGTAATGAAGAATTACATGAT 9760

N Y V G M R Q W Y N D P S G G Y P D L G N E E L H D

9761 GTTTTATTTAGCTTAGCTTACAACTGTCTCCATTTTTATAAGAGTCTACCCATTGTTAGACAGAGATTCAAATGTTATTT 9840

V L F S L A Y N C L H F Y K S L P I V R Q R F K C Y L

9841 AGAACCTAGCTTTCAGAGAGTAGGAATTTATAACATCTGTTTTCAATGTAAACAATGTTACAAAGTGATAATTGAGTCAA 9920

E P S F Q R V G I Y N I C F Q C K Q C Y K V I I E S K

9921 AACCAGTGAAATATGATCCCGAGGTGAAATTCTTCACCCTCCTCCTATCAAACTTTCAAAAGAGTAGTTGGCGTAGATCC 10000

P V K Y D P E V K F F T L L L S N F Q K S S W R R S

Bel2 ORF

10001 AGAATGTGTAGACACCTAGAGTGGCATGAGAACGAACAATTCTCTTATAGATCTACGCGACCTCGAGAGCAGGGTTCGGA 10080

R M C R H L E W H E N E Q F S Y R S T R P R E Q G S E

M R T N N S L I D L R D L E S R V R S

10081 GCCTTCCCCCGCTGCCACCAGCAGCTGCTCACGAATGGATTCGGTCCCAGATCCTCCACGAGGACCACGAGAGTGTCTCC 10160

P S P A A T S S C S R M D S V P D P P R G P R E C L Q

L P P L P P A A A H E W I R S Q I L H E D H E S V S

10161 AGGGGCCTTCAATGTCAGATTGTGCCAGTCCTATCCCCGGTCCCTGTACCAGCCAGGAGCCCTATGCAGATGTTGATGCT 10240

G P S M S D C A S P I P G P C T S Q E P Y A D V D A

R G L Q C Q I V P V L S P V P V P A R S P M Q M L M L

10241 TTTCTGGAAGGGCTACTGCAACAGCCACAAAAAGGACGAGCCGATTCCGGAATGGGTCTGGACCCCGGAGCCGGCTGGAC 10320

F L E G L L Q Q P Q K G R A D S G M G L D P G A G W T

F W K G Y C N S H K K D E P I P E W V W T P E P A G P

10321 CGAGGATTTGGAACGCTTCATCCTTGGTGATGACAACAGCTGTGCCAGGGATTGGACTGATTCAATGCACTCTGACAGCT 10400

E D L E R F I L G D D N S C A R D W T D S M H S D S *

R I W N A S S L V M T T A V P G I G L I Q C T L T A

Bel1 ORF end

10401 AATTCCTGTAAGGTTCATGTGTGTGGGGGAAGAAGGGATCCCTGGTATGTGGGGACTTGTGGCCCCACTGTATGCTGGAA 10480

N S C K V H V C G G R R D P W Y V G T C G P T V C W N

10481 TATGTATTATACAGGAGACCCTTTCAGGAGGGAACAACATGAGCCCACTTTGGTTCATCTTGCCCCTCATCATGATAATC 10560

M Y Y T G D P F R R E Q H E P T L V H L A P H H D N P

10561 CCTTCCTCTGTGGAAGGAGGCCTCATATTGATTTCTGTAAATCCTTCAGTTATGAAGGAGATAGCCCCCTCCAACAGAGT 10640

F L C G R R P H I D F C K S F S Y E G D S P L Q Q S

10641 ATTAAGAAATACAAAGGCTTGACCACTGAAGTAGTCATGGGAACGGAACCATTGGCAACCACTGTCATTGAACTTGAATG 10720

I K K Y K G L T T E V V M G T E P L A T T V I E L E C

10721 CATGGAATTCCAGTTGGCCTCCAGGGATGTTCCTGGAACTCCAACTTTTCCAAATTGGGGATTCAGTCCTTGGGGATACA 10800

M E F Q L A S R D V P G T P T F P N W G F S P W G Y T

PPT **3'-LTR**

10801 CCCTACATATGATGGCCTTCGGGCCCGAGGGACCAAACACC**AAGGAGAGGGTG**TCATGGGAAAAAGGTCTCTTGACTAGG 10880

L H M M A F G P E G P N T K E R V S W E K G L L T R

10881 AATTTTAGCTACTTTAGATCTAGTGCTGTGAGACCACAGGAATTGGAAGCTATGGTAAATTCCAAATTGGTATTGGCCCC 10960

N F S Y F R S S A V R P Q E L E A M V N S K L V L A P

10961 TCTTCATCCCTTCCAGTACAGAGCTGGTATACAGCTCTGGGTTAATACAGCCTTGCCCCCCGACTGGGTTGTACTGGATC 11040

L H P F Q Y R A G I Q L W V N T A L P P D W V V L D P

11041 CCAGAGGAAATAGGTGGGTGTCTTCAGGACAAGGCTGGGAACAAGTGTATGAGGACATAGTGGACTGTGCCGACCCTGAA 11120

R G N R W V S S G Q G W E Q V Y E D I V D C A D P E

11121 CACTTCACCTCAGGTGATCCAGGTATGACATTGCCTCTGCATATCATACCGGAGGAATGTAGGGATGATTATGAGAGTGA 11200

H F T S G D P G M T L P L H I I P E E C R D D Y E S D

11201 CGGGGAAATACCACGTCACCGAGGCCTTGGCTGGGTATCAGATCCTGAAAGCCCCAGGCCTCCTAGTCCCTCCAACAGCA 11280

G E I P R H R G L G W V S D P E S P R P P S P S N S T

11281 CTATGTCGGCTTCGCCACTAGATGGAGCCTGCTCCGAAAAAGAAGACTGGGAGGAACCACCTCCTCTCAGACACAGGCCT 11360

M S A S P L D G A C S E K E D W E E P P P L R H R P

Bel2 ORF end

11361 TCCACTCCTTATCCACCTCCTCCTCCTGAAAGTAATGAAAGTGAGGAGGATTAAGGGATTAGTGCTGTAGCATATTGGCA 11440

S T P Y P P P P P E S N E S E E D *

11441 CAGTCCTAGTTGAGGGTTACGCAGCCCACTTGACTCTGGCAGTTCGAATGACTCAAGTGGATGTGTAGCCTTTTGCCAGC 11520

11521 AATTGCAGTATTACCACATATAGCTTTGCTACATATTGTTATTTGCCATAGTACTTAAGGGATTAATGGTCTAGCAAGAT 11600

11601 CACTAGGAGCTAGGCTAGCAATCCAGTTAGACCATTAGTCACTTGACATTCACACTGCAAATGTGAGTTCGAGTCTTTGT 11680

11681 AGTGTGGGGTGTTGAGTTTACCAGCTTTTGCTAATGGAAATCCATACATGGTATTGGTACCGATAATTGTATTTCCAATG 11760

TATA box **U3/R boundary**

11761 ATTGTATTAATGTTTTAG**TATATAA**GGATCCCAAATAGATTGTACGGGCCCCCACTCACTTCAGCTGGCTGCGACCAGTG 11840

11841 TGAGATTGGTGTCTCCAGACTTGGTAAGAGATTTTTATCATTATATTGTAAGGCTTGGATAATTAATTGTTAGATGCTCT 11920

Polyadenylation signal **R/U5 boundary**

11921 GCTATTAAT**AATAAA**TTGGTTGTATTTTGAGAACCACAATTTAACTATTGTCTCTCTTTATTCCTCCTGGTTGAGGACAA 12000

12001 GGGAACCTCTCCCTCTCGGGTCTTGCAGTAATAAATAAGTATAAGGGGGAGACTAAGTCTCTGACATATTATAAAAATAC 12080

12081 TTATTTATAAGATCCTTTGAGAGGGTGAGTCCACGA**CA**

PSFVaye

5′truncated 5′-LTR

1 AAACTTTCTAAATCAGATTGCTGGGTAATAATTCATAACATCCTCAGTTTATACTGAGGAAACCTTGATTAAATTTACCT 80

PBS

81 TGAAGACTTTCTGGTAGTGAAACCCACAG**CA**AT**TGACACCCAATGTGGGGC**TCGAAAACCTAGGCACTAATTTTGAAAGT 160

Gag putative RF

161 GGCATATCCCTAGGGGCTCTAGACCAAGGTCCAGAGAATGGCTCAAGATAACATAGACCCTCAAGGTTTAGCAGCTTCAT 240

M A Q D N I D P Q G L A A S Y

241 ATTTAAATGTTGGAATTAATGTTCCCTCAGCCAACAATAATGAAATAGCAGTCCAAATGACTGCTGGTCCACGGG-AGTT 320

L N V G I N V P S A N N N E I A V Q M T A G P R ? V

321 GGAGATTAATGGCAGAGAATTTGTTTGGCCTTTCAATACCAAAATGGTCAACCTTCACCAGTTCCCCCATAGAGGCTTAT 400

Cytoplasmic retention signal

G D * W Q R I C L A F Q Y Q N G Q P S P V P P * R L I

401 AGCAGAACCAGTGAATCTTCAAAATGACTATTTAGTAGAAGCATCTCATAATTTATTAATACAAACCTTTCATAACATTG 480

A E P V N L Q N D Y L V E A S H N L L I Q T F H N I A

481 CACCTGGTGTTAAATGACATGGACCTCTATCAGACGGAAATTATCAACCTGGAGATGGGATCTCTGAAGATTATGTACCT 560

P G V K * H G P L S D G N Y Q P G D G I S E D Y V P

561 ATATCAGATCAAGAATTATTACAAGTAACTGCTCTAGCAACTGCAAGAGCAGAAATCAAATTACAGAGGCAAAATTTTAA 640

I S D Q E L L Q V T A L A T A R A E I K L Q R Q N F N

641 CTGAACTATGGCTGCTAATGTTCAAGGAAGACCCTTGCTGCCTAATGTTGGGACAGGACCACAGATTCCTCAAGGTATTC 720

* T M A A N V Q G R P L L P N V G T G P Q I P Q G I P

721 CCATTAATCATATAACAGCAGTTATTGGACAGAGTCCTGAAAACCCTGAGGAAATATCATTCTGGTTAGGCAAAAATAGA 800

I N H I T A V I G Q S P E N P E E I S F W L G K N R

801 GCAGGCCTTACAGGAATCTTCCCTGTGAATAATCCAAATATAAGACTAAGGGTGATTAATACCTTTGTAGGATCTCATCC 880

A G L T G I F P V N N P N I R L R V I N T F V G S H P

881 TACCTTGATATTGCAACAAGGAGAAGCTGATACCTGGGAAGACACTATTGTTAACCTACATCAGAGAGCTCACGGAGCTG 960

T L I L Q Q G E A D T W E D T I V N L H Q R A H G A V

961 TGGCCAGACATAGGCTGCCAGGCCTCATCACTGTTATCTTCAAAAAGGAAGGAGTCCAGGTGGCTTATAGCATGGGCCTA 1040

A R H R L P G L I T V I F K K E G V Q V A Y S M G L

1041 ATGCTTTCAAAAGACTTCGATTTAGTGTGGGGTGTGATTGGGGGTTTACTGTCTGGTCAAGCCCTGGTTACAAATATACA 1120

M L S K D F D L V W G V I G G L L S G Q A L V T N I Q

1121 AGGACAATGATACCTTTTACCTAATACATGAGCAAGACAAGAACAGTTTCCAGGTGTAGTTTGAGCTACATAAAATTTAT 1200

G Q * Y L L P N T * A R Q E Q F P G V V * A T * N L L

1201 TGGGATTAGATGTTTTTGGAAAGTCTATGAGACCTCCCGCAGTACAAAGACCTCCTCCTCATACTCAAGGTAACAGTATG 1280

G L D V F G K S M R P P A V Q R P P P H T Q G N S M

```
1281 GGTAACAGAGGAGGAGGAGGAGGAGAATGATCTCAGCCCAGGAGACAACAGAACCTACAACAGGATCACGATGAACGCTG 1360
                          Glycine-arginine box I
     G  N  R  G  G  G  G  G  E  *  S  Q  P  R  R  Q  Q  N  L  Q  Q  D  H  D  E  R  C

1361 TGAAAATCCCCAGGGTGAAAATGAAAGTAACTACAATGATTAAGGGCAACCACCTCAACATGGATATAATTTAATCAGAA 1440
                                                          Glycine-arginine box II...
      E  N  P  Q  G  E  N  E  S  N  Y  N  D  *  G  Q  P  P  Q  H  G  Y  N  L  I  R  N

1441 ATCCTCAGGCCAGGCGTGGTGGCTCATGCCTGTAACCCTAGCACTCTGGGAGGCTGAGGCAGGAGGATCCCTTGAGCTCA 1520

      P  Q
                                                              SINE
1521 GGAGTTCAAAACCAGCCTGAGCAAGAATGAGACTCCATCTCTACTAAAAAAATAGAAAAAGTTAGTTGGGCACAGTGGCG 1600

1601 CATGCCAGCTACTCGGGAGGCAGGAGGGTCACTTGAGTACAGGGGTTTGAGGTTACTGTGAGCTACGCTGATGCCACAGC 1680

1681 ACTGAGCAACAGAGCAAGACTCTGACTCAAAAAACAAAACAAAACAAAACAAAACCCAAAATCCTCAAAGTCCCAATTGT 1760

                                                                            S  P  N  C

1761 TATGGCAGAGGAGGCAGAGGGCAAAATCCTTATCGACGAAATGCTGAACAGCCTGAAGAAAATTCTCCTGATGTCTCTCA 1840
     ...Glycine-arginine box II
     Y  G  R  G  G  R  G  Q  N  P  Y  R  R  N  A  E  Q  P  E  E  N  S  P  D  V  S  Q

1841 ACCTTCTGCAGGCTGCAGAAGAGCAGGAGGACCTGCTTGGGTAGTTAAACTGATCCGTTGCTCCCCGAGTTCCTCCCCGC 1920
             Glycine-arginine box III
      P  S  A  G  C  R  R  A  G  G  P  A  W  V  V  K  L  I  R  C  S  P  S  S  S  P  P
                                 Pol putative RF                 end Gag putative RF
1921 CACAGCCTCCAGAGCCCAGCATGCAAGACAAGCGAGTAGCTGTAGCGATCAACAAATAACAGCTTTAAGAAAGTTATGCT 2000

      Q  P  P  E  P  S  M  Q  D  K  R  V  A  V  A  I  N  K  *

                        M  Q  D  K  R  V  A  V  A  I  N  K  *  Q  L  *  E  S  Y  A

2001 ACTTTGGCAAAAATTACATGCTCTCCTTCCTGTCTCCTAGAGCCCTACCAACCAACTAGTGACACTTTCATTGAAACAGT 2080

     T  L  A  K  I  T  C  S  P  S  C  L  L  E  P  Y  Q  P  T  S  D  T  F  I  E  T  V

2081 TCATGGACAAAAAACACTTCCCCTCTATTACATTGATCACAGAGTAGAGGGACAAAAAGTTACTTTTGAAGTCATT-CCA 2160

      H  G  Q  K  T  L  P  L  Y  Y  I  D  H  R  V  E  G  Q  K  V  T  F  E  V  I  ?  N

2161 ATGAGAACAATATTATTATTTTGTCCCCTATCAACATTCCTTGGATTAAAGTTAAAAGACAACAATTAGA-ACTAAGAGA 2240

      E  N  N  I  I  I  L  S  P  I  N  I  P  W  I  K  V  K  R  Q  Q  L  ?  T  K  R

2241 ATTGATATAGAAAAACAACAGGAAACTTTAGTTAGATTTTCCAAGGTTTCTGAAAAAGGGAAACAGAAACTGAAGGATCT 2320

     I  D  I  E  K  Q  Q  E  T  L  V  R  F  S  K  V  S  E  K  G  K  Q  K  L  K  D  L

2321 CGTCCTCACCTTTGAAAATCCACAGCAACAATGGGAAAATCAAACCGGTTACAGGAACATTCCTCCTCATAATATAGCTA 2400

      V  L  T  F  E  N  P  Q  Q  Q  W  E  N  Q  T  G  Y  R  N  I  P  P  H  N  I  A  T

2401 CTGGTACAACTCCTTCAAAACCTCAAAGACAATATGCTATTAATCCAAAAGCCAGGTCAAGTATTCAAGCAGTCATAGAT 2480

      G  T  T  P  S  K  P  Q  R  Q  Y  A  I  N  P  K  A  R  S  S  I  Q  A  V  I  D

2481 GATCTATTAAAACAAGGTGTTTTGACTAAATAAACAAGTGTTATGAACACTCCCGTATATCCAGTTCCTAAGCCTAATGG 2560

     D  L  L  K  Q  G  V  L  T  K  *  T  S  V  M  N  T  P  V  Y  P  V  P  K  P  N  G

2561 CCAGTGGAGACTTGTGCTAGATTATGAGGTTGTCAATAAGCCAAATCCTCCACTTACAGCTCAGAATTTTTGCTCCATTG 2640
```

Q W R L V L D Y E V V N K P N P P L T A Q N F C S I G

2641 GGCTTTTGACTACCTTACCTAAACATAAATATAAAACTACTTTAGATTTATCCAGTGGTTTTGAGGCTTACCCAATTACT 2720

L L T T L P K H K Y K T T L D L S S G F E A Y P I T

2721 GAAGAAAGTCAATGGATT-TTCCTTGGTTAGGTTAT--GAATGCTTGGACCAGGCTACCCCAGGGATTTTTAAATAGTCC 2800

E E S Q W I ? P W L G Y ? N A W T R L P Q G F L N S P

2801 TTCCTTGCTTATAGCTGATGTTACAGAGATCTTAAAGGACATCCCTAACATGGTAGTCTATACGGATGATATTGGCTGGG 2880

S L L I A D V T E I L K D I P N M V V Y T D D

2881 CATGGTGGCTCACGCCTGCAATCCTAGCGCTCTGGGAGGCCAAGGCAGGAGGACTGCTTGAGCTCTGAGTTGGAGACCAA 2960

SINEs

2961 GAGCGAGACCCTGGCTCTACAAAAAACAGAAAAACTAGCCAGGCATTGTGGCACATGCCTTTAGTCCCAGCTACTCCAGA 3040

3041 GGCTGAGGCAAGAGGATCACTTGAGCCCAGGAGTTTGGGGTTGCAGTGAGCTGTGATGACGCCACTGCACTCTACCCCGG 3120

3121 GTGACAGAGAGAGAGAAAAAGAAAAAGAATTGTTATATGGCAGATGTGTATTTAAGTCACCAATCTGAAGAACAACACTT 3200

V Y L S H Q S E E Q H L

3201 GGAAGCCGTGGGAAAAAAAAATCTTTGACAATTTGTTAAAATCTGGGTATATCGTCTCTGTGAAGAAATCAGAGATGGCAA 3280

E A V G K K I F D N L L K S G Y I V S V K K S E M A M

3281 TGCGTGAGGTTACTTTTTTAGGATTTACCCAAGAAGGAAGAGGACTTATGGAAACACATAAAGAAAAATTACAACTCAAG 3360

R E V T F L G F T Q E G R G L M E T H K E K L Q L K

3361 CCACCT-AAAATTTAAAAGAGTTACAAGGTATATTAGGCCTTTTAAACTTCACAAGGAACTTTATTCCAGATTTTGCTGA 3440

P P ? N L K E L Q G I L G L L N F T R N F I P D F A E

3441 ACTTACACACCCCCTTTATGATCTAATCAGTACAGTTGGTACCAAGATGATTCACTGGAAACTGCAATATGACCTTATTC 3520

L T H P L Y D L I S T V G T K M I H W K L Q Y D L I L

3521 TCTCAGAACTTATAAACAAATTAAATTCTTCTGTTTTCCCAGAGGAAAGAAAACCAGATATTTCCCTTATTAAAAAAGTG 3600

S E L I N K L N S S V F P E E R K P D I S L I K K V

3601 TACATTTCCAAAATTGCTGGTTTTGTAAGATTTTACAATTATATTAGGGAAAAAAAAC--ATCTCTCATTTAAATATAGT 3680

Y I S K I A G F V R F Y N Y I R E K K ? I S H L N I V

3681 ATTCTCAAAAACTAAGCTCAAATTTAAGAATATTGAAAAGCTTCTAACTACTATACATAAGCCCCTTTTAAAAGCCTTTG 3760

F S K T K L K F K N I E K L L T T I H K P L L K A F D

3761 ATATGGCTCAAAGTCAGAACATTAAGATTTACTCACCAATAGCATCTCTAGCAAGGATTCAAAAAACTCCAATACCAGAA 3840

M A Q S Q N I K I Y S P I A S L A R I Q K T P I P E

3841 AGAAAGGCCTTCTCCTCTAGATGGACTATATGCCTGGTATATACTGAAGATCCAAGAATCTCTTTTTATTTTGATTCTAT 3920

R K A F S S R W T I C L V Y T E D P R I S F Y F D S M

3921 GCTTCTAGACCTTTCTGATTTACCTGAAATTGAAACTTCTTCCATGACTCTTTCACCCCTTCAAGATTACAAGTATGTTT 4000

L L D L S D L P E I E T S S M T L S P L Q D Y K Y V F

4001 TTATATG-GATAGATCCACCATAACTTCTCCTAAAAGAAAAAAGAAAA--AAACACTCAGCAGGAACAGGAATTATTCAG 4080

I ? D R S T I T S P K R K K K ? K H S A G T G I I Q

4081 GCAACATTTTCTCCAGATTTCACTTTTCTAAAACAATGGCAACTCCCACTGGGAGACCACACTGCTCAATTTGCTGAATT 4160

A T F S P D F T F L K Q W Q L P L G D H T A Q F A E L

4161 AGCTGCCTTTGAATTTGCCATCAAA--AAAGACTACTATGCTTAACAAGCCCGTATTAATTGTTACCGACAGTTTTTGTA 4240

A A F E F A I K ? K T T M L N K P V L I V T D S F C I

4241 TCTCAAACTCCTATAATAATGAACTTAAGTACGGG-CCCCTAATGGATTTCTTACTGCCAAAAGAAAGCCTCTTAAACAT 4320

S N S Y N N E L K Y G ? P N G F L T A K R K P L K H

4321 ATTAGTAAATGGAAACAAATATATGATATTGTCAAGGACTGTATCACTGAAATTCACATGATTCACGAGCCAACCCATCA 4400

I S K W K Q I Y D I V K D C I T E I H M I H E P T H Q

4401 AGCTGACAGTGCCACTCAGCATTCCTTTGGCAATAATCAGGCTGATAGATAGCTACACAAGCCTCTTATAAAATTTTTGT 4480

A D S A T Q H S F G N N Q A D R * L H K P L I K F L F

4481 TCT--AAAGATACAACAAGCCTGGATAAGGTGCT-AAGCCAATCCTAGATAATACCAAACCTAATCCACCCGGTTATCCT 4560

? K D T T S L D K V ? K P I L D N T K P N P P G Y P

4561 AATAAATATATGTGTGAATTATGTAATAACAAAGTTTATGTAACCCTGCCTAAAGGGAGAAGAAAAATGCCCCCAGTCCA 4640

N K Y M C E L C N N K V Y V T L P K G R R K M P P V H

4641 TTTACAATTACAATTGGCTCAAAAGGCACATAATTCTTTAAGAAACCTACATGGAGGAAGAAACACTACTCTTTTAAAAT 4720

L Q L Q L A Q K A H N S L R N L H G G R N T T L L K L

4721 TACAAAATTGCTATTGGTGACCTAATATGCTGAAGACAGTCAAGACAGTTCTCAAAGACTGTGAGCCTTGTTTAATGGTC 4800

Q N C Y W * P N M L K T V K T V L K D C E P C L M V

4801 AACTTTAATAATGTTAAGACAATTCCTCCTATTAAAATTCAACATGCTCAAAATCCTTTTGATAAAATTTATATGGACTA 4880

N F N N V K T I P P I K I Q H A Q N P F D K I Y M D Y

4881 CGTAGGACCTCTCTCTCCTTCCCACAGGTATGAACATGTTTTGGTACTTGCTGATGCTTCAACTAGGTTTTGTTTGTTAT 4960

V G P L S P S H R Y E H V L V L A D A S T R F C L L Y

4961 ACGCCACTGATGTTCAAACTACTGATGTAACT-TGAAAGCTCCCACCCACTTCTCCAGTGTTGCAATTCCACGGGTGCTA 5040

A T D V Q T T D V T ? K A P T H F S S V A I P R V L

PPT

5041 TACTCTGATCAGGAGTCAACATTTACCTCTTCCAGCCTTGGCCTCTGGGCC**AAGGACAAAG**GAGTACCGCTTGAATTCAG 5120

Y S D Q E S T F T S S S L G L W A K D K G V P L E F S

5121 TACTACGTATCACCGCCAAAGTAGTGGAAAGCTGAAAAGGGCAAGTGGCAAAATAAAATGAGCTCTCACTAAACTACTAG 5200

T T Y H R Q S S G K L K R A S G K I K * A L T K L L V

5201 TTGGTAGACCCAGAAAGCGGTATCGACTCACCCTGGAAGTACAACTAGGTATTAGTAATATTCCTAATCAGCAGGGCAAA 5280

G R P R K R Y R L T L E V Q L G I S N I P N Q Q G K

5281 ACCCCTTATAAGCCTCTTTTTGGGGTTTCCCCAAATGTTTTTTTTTCCAAGGGAACTGAAATTTCCAGAGAGGAACAACA 5360

T P Y K P L F G V S P N V F F S K G T E I S R E E Q Q

5361 GGCCGTCTTACAAGAACTATGTTCATATCTATTTCCTATATTACCACCTGTCAAATCTTCAAGAG--TCGACCCCACAGG 5440

A V L Q E L C S Y L F P I L P P V K S S R ? S T P Q V

5441 TTGGCCTTCTTGTCCAGGAGAGGATAACCAAGCCTACAGGTTTGAGAACAAGGTGGAAGAAACCCACTCCCATAATAGAA 5520

G L L V Q E R I T K P T G L R T R W K K P T P I I E

5521 GTTCTGTCAGACCAAGTGGTGAAGATATTGGATGAACAGAACCAACCTTAAACTGTGAGTACAGATAACCTAAAATTAAC 5600

V L S D Q V V K I L D E Q N Q P * T V S T D N L K L T

3′truncated Env putative RF

5601 AGCTTTTCAAGATGGACTCTCACATGACACTGACTTAGTGGCTAGCCTGGAAACAGAAAGAACTACAGATAATAAGAAAA 5680

A F Q D G L S H D T D L V A S P E T E R T T D N K K T

WXXW motif

M D S H M T L T * W L A W K Q K E L Q I I R K

end Pol putative RF

5681 CTGCTTCCAGAAGTATCTACTGATGTTGAATCTTTGAAAACACCTAATTTAGAAAA-AGTAATTTTTGCCTTAAAATGAG 5760

A S R S I Y * C * I F E N T *

L L P E V S T D V E S L K T P N L E ? S N F C L K M R

5761 GTACTGGGCATATGTGATCTGTGCTATTATTTCCAGAATATTGATTTGGATCATGTTGTTTTTAATATTATTTTCAGCTA 5840

Hydrophobic transmembrane region with

Y W A Y V I C A I I S R I L I W I M L F L I L F S A I

5841 TCCTTGTTTCTACACTTATAGCTGTTTTTACATTACAATGGAATAAAGCAATATATGCTCTTGGACCTATAATAATATGG 5920

a signal-peptide peptidase cleavage site

L V S T L I A V F T L Q W N K A I Y A L G P I I I W

5921 AACCACTCATCTGCACAAGATTTAACCTATTTTTCACAGGCTCATGTATGAAAGTCTAGGGCTATACATTTTCAATTATC 6000

Furin-cleavage site

N H S S A Q D L T Y F S Q A H V * K S R A I H F Q L S

6001 TA--GTAGATATTGATATTTAAGCCCTACCTCAAGGTATCTTTATGGAACCTTATCCTAAACCTACTGTGGCCAAGGAGA 6080

? V D I D I * A L P Q G I F M E P Y P K P T V A K E R

6081 GGGTCCTTGGCCTTTCTCATAGGGTTTTAGTTGATTCTTCTGCTCTATTCCAGGATGCTAAAATACTCAATGATCAAAGT 6160

V L G L S H R V L V D S S A L F Q D A K I L N D Q S

6161 AAGTTTCTATTAGTAAACAAGATTAATGAGGAAATGAG-AGTTTACAAGACTTGGTACTGCAATTTGATTTCCCACCTGA 6240

K F L L V N K I N E E M ? S L Q D L V L Q F D F P P D

6241 TCATCCTAAAACTCAAAGACAGTATGTTGAGCATAAATGTTGTCAATGCTTTGCTCACTGTTATGTCATAGATTATGGAG 6320

H P K T Q R Q Y V E H K C C Q C F A H C Y V I D Y G V

6321 TTGACAGGCAGTGGCCTACCAAGGAAATTATTCAAGATCATTGTCCTTTACCCTCAGTTCCTTATTCTCAGGTTCCCCTT 6400

D R Q W P T K E I I Q D H C P L P S V P Y S Q V P L

6401 ATG-CAGCCCAAGCTGCATGGCATTACTTTTTAAAGGTAGAAAGAATTAGGCCGAAAGATTGGACTGAAGAAAAATATCA 6480

M ? A Q A A W H Y F L K V E R I R P K D W T E E K Y Q

6481 AAAAGGAGCCAGGACTGAAGCCTTCAGAGTACCAGGTGAAAATGATCACAAGGAGAAACAGGCTATTTTTTGTTCTCCTG 6560

K G A R T E A F R V P G E N D H K E K Q A I F C S P D

6561 ACCTGTATTAAAATTGGTGGAATAACTATCTCAGTCTCTCAGAG-ACATAAAATTTTTGAAGATAAAATTGAAGTCC-TA 6640

L Y * N W W N N Y L S L S E ? I K F L K I K L K S ?

6641 TCGAAAACAGGAAAATCAGAATTTAAAAATGCAGCTCTTCCTCCTAGGTAGAATATGAAAGAAGGTGAAACATTTAAAGA 6720

S K T G K S E F K N A A L P P R * N M K E G E T F K E

6721 AACTCCTTTGACTGATTTTTGCAATAAGCCAGAACAAGTACTCTTTATTAATAGCACGTATTATACCCGGTCCTTACGGG 6800

T P L T D F C N K P E Q V L F I N S T Y Y T R S L R E

6801 AAGGAGATTGCACTTTCACTAAGGATAACGTAACGCATTACCCACTCTGCAAGAATTTCAACAGGAATCTTAGAGATCAT 6880

G D C T F T K D N V T H Y P L C K N F N R N L R D H

6881 CCTTATGTCTGTAGGCACTTGAGACTGAGAAAAGATGAAGAAAAAACAGTGTGTCCTGATGAAAAAACAACCACCAAATG 6960

P Y V C R H L R L R K D E E K T V C P D E K T T T K C

6961 CTTATATTATCTTAAGTACTTTTTCATGGAAGTATTTACTAACTTTGGATACTTAGCATTCCACAATGCTTTTCCAGCTC 7040

L Y Y L K Y F F M E V F T N F G Y L A F H N A F P A P

7041 CCATTTGCATAGAAGCCAAGAAAATACAGGAACCTAAATATAAACTAACC--TGTTTATGAAAAATGTGTCACTCAAGGC 7120

I C I E A K K I Q E P K Y K L T ? V Y E K C V T Q G

7121 CTGAAACATGACTTATCCTCTGTATTACAAAATTTACAAAAAATACTAACTGAAAACATCAGATAA--CAATACACGAAT 7200

L K H D L S S V L Q N L Q K I L T E N I R * ? N T R I

7201 TTTTATAGGAAATGAAGCCAAGTAAAGAGATTTTTGGGGTACAAAGTCAATCAATCTTACACAAATTAAATATTATGAGA 7280

F I G N E A K * R D F W G T K S I N L T Q I K Y Y E I

7281 TAAAAAAAATTTCTCTAAA-GAGAAATTCTGTGTAATAAAGCAAAGCCCAGAAGTGTTACGCTGGTGGAACAGGGTGGTG 7360

K K I S L ? E K F C V I K Q S P E V L R W W N R V V

7361 GATTTGAGATTCTGCTTGCCACACATTTGGAAAATGCAGGGGAAAAGAAGTCCTTCTGCGGAGTGTTAAAATAACTAAAT 7440

D L R F C L P H I W K M Q G K R S P S A E C * N N * I

7441 AGTTGGCAATTAGACTGAACTGAGTCTAGTGCCCTGGGTTCCTACCTAAGAACACCAAAATCTAACTCAAATGCATTTTT 7520

V G N * T E L S L V P W V P T * E H Q N L T Q M H F F

7521 TTGGTAAATTACTAACTTAGGGAGAAACAAAATTTAGGCTTAACCAACTACAAACCACCAATTAACCTCTGCTATATAAC 7600

G K L L T * G E T K F R L N Q L Q T T N * P L L Y N

7601 CAGAAAACAGCCACCTGGAGAGTCCGAACAAGGCGACTACACAGCTGCAACCAAGCACCCGCTGGATTGGGTTTGCTCCC 7680

Q K T A T W R V R T R R L H S C N Q A P A G L G L L P

7681 TCGCGCACCTTACGAAACGCCTTGCCTTCAGGCCCCTCCCACGGACCCCAAACACAAGTCTCGGCTGGGGCTTCCTGAGT 7760

R A P Y E T P C L Q A P P T D P K H K S R L G L P E S

7761 CGTGAATCTGTTTGCTCTAACAAACTCTCTCATGTGTCAACAGTGCCTTAATTTACTTTTTAACAGGAGAGAGGAGGGAC 7840

* I C L L * Q T L S C V N S A L I Y F L T G E R R D

7841 GGGGACGGGGACGGAGTCGGGATCCGC

G D G D G V G I R

ChrEFV

5′ truncated Pol putative RF

1 CCTATAAAACAGAAACAAAACCATATTAACCATAAAGCTACTCCATCTATACAAGTTATAGATAATCTTCTTGCTCAGCA 80

P I K Q K Q N H I N H K A T P S I Q V I D N L L A Q Q

81 AGTCTT-AAAAAACAAACCAGTCCTATGAATACTCCTGTGTATCCAGTCCCCAAATCAGAAGGAAAATGGAGAATGGTCC 160

V ? K K Q T S P M N T P V Y P V P K S E G K W R M V L

161 TAGATTATCGTGCTATCAACAAAGTTATTGAACCTATTGCTGCACAAAATTCTTTCTCTACTAGCATTTTGGCCCAGTTA 240

D Y R A I N K V I E P I A A Q N S F S T S I L A Q L

241 CCTAAGAAAAAGTTCAAAACAACTTTAGACTTAAGTAATATATTTTAGGCAATACCTATTCATCCCAATGACTACTGGAT 320

P K K K F K T T L D L S N I F * A I P I H P N D Y W I

321 CACTGCTTTCACTTAGAAAAGTCTGTAACACCATGTCTGGACAAGACTCCCACAGAGTTTCATCAATATCCCAGCCCTCT 400

T A F T * K S L * H H V W T R L P Q S F I N I P A L F

401 TCCCAGCAGATATTCAACAACT-TTAAGTCCTCTACCTGATGTAATCGCATATGTAGATGACATCTATTTCACTGCAGAT 480

P A D I Q Q ? L S P L P D V I A Y V D D I Y F T A D

481 ACAGAGCAACAACAGTTACAACAAATCCTATATATCCTAGGAAATGCAGGATGTATCTTCTCTCCCAAAAAGTCAGCCAT 560

T E Q Q Q L Q Q I L Y I L G N A G C I F S P K K S A I

561 TGGGAGACATACAGTTCCTTTCTTAGGTTTTGAAATCACAAAATAAGGCCGAAGCCTAACAGAAGATTTCAAAACTAAAC 640

G R H T V P F L G F E I T K * G R S L T E D F K T K L

641 TT--AAATATTAAAGAACCAGGCAGCTTAAAACAACTGCAAAGTGTATTAGGACTTTTCAACTTTGCTAGAAACTTTGTT 720

? N I K E P G S L K Q L Q S V L G L F N F A R N F V

721 CCTGATTTTGCTGTTTTAACTGAACTTTTATGTAAACTTATTTCCACCGCCAAAGGTCAACATACTCAATGGAATGGAGA 800

P D F A V L T E L L C K L I S T A K G Q H T Q W N G E

801 AGCAGCTGCAGCATTATCAACTTTAATAAATAGTGTTAACAAAACAAAGTATTTAGCAGAATGAAATGTTAAA-CTCCCT 880

A A A A L S T L I N S V N K T K Y L A E * N V K ? P S

881 CTATGGTCAAATGCTATGCAAGTCAAATGGCTGGTTTCGCAGGACTTCATAATGAGTCAGAAACCACACCCATTTCCTTT 960

M V K C Y A S Q M A G F A G L H N E S E T T P I S F

961 CTGTCTTTCATATTCACTCCAGCTGAGACAAAATTTGCTCCCACTGAAAGAATATTAACTATTATACATAAGACTCTATT 1040

L S F I F T P A E T K F A P T E R I L T I I H K T L L

1041 AAAAGCTTCTGACCTCGCATAAGTAAGCATAATAAAAATTTACTCACCTGTAACACCACCAACTAAACTTCAAAAGATTC 1120

K A S D L A * V S I I K I Y S P V T P P T K L Q K I P

1121 CATTAAGTGAACGCAACGCCTTAAATTCCAAATGGATCACTTGGCCCTCTCACTTTAAAAATCCACAGCTGTAGTTCATT 1200

L S E R N A L N S K W I T W P S H F K N P Q L * F I

1201 TATGATCCCAATCTGCCAGATCTCTCTCACTTACCAGCACCTGCAGCACAGACTTTGCTACCATTAACTGAATATACATA 1280

Y D P N L P D L S H L P A P A A Q T L L P L T E Y T Y

1281 TGTATATTATACTGATGGATCTGCAATCAAGTCCTCCAAAATTAATTTTCAATACTCCTCTTGGACAGGAATTATAAAAG 1360

V Y Y T D G S A I K S S K I N F Q Y S S W T G I I K G

1361 GCAAATTCACTCCTTCATATACTGAAGTGAATAAATGGTCTTTCTCAGCAGGTGACCACCCAGCACAGTATGCTGAAGTC 1440

K F T P S Y T E V N K W S F S A G D H P A Q Y A E V

1441 ACAGTTTTTATTTTTGCTTACAAAGATGCCTCAAAACAAGTAATGCCTGTCTTAATAGTTACAGATAGTGACTATGTCTT 1520

T V F I F A Y K D A S K Q V M P V L I V T D S D Y V F

1521 TAAAGCTTATACTAAAGAATCACAAATACGGATTTCTAATGGTTTTCATAATGCAAAACATAAACCTATAAAACGTATTG 1600

K A Y T K E S Q I R I S N G F H N A K H K P I K R I V

1601 TTAAATGGCAGCAAATAACTTCATTGTATGTGCCTAAGAGTCACGTAATCCATGAGCCAGGGCACCAGACTCCTGAT--C 1680

K W Q Q I T S L Y V P K S H V I H E P G H Q T P D ?

1681 CTACAAAGGCCCACTACTGGTAATACCATAGCAGACCCCCTGGCTACAAAAGCTAGTAGAGAGGTAAATGTTATAGAGAA 1760

L Q R P T T G N T I A D P L A T K A S R E V N V I E K

1761 ACCCTAGACTGAGAATAAAGACTTGAAACAAGTAAAAGATTTACAGCAGGGTAACAGTAAATATACTGTTAAATATCGAT 1840

P * T E N K D L K Q V K D L Q Q G N S K Y T V K Y R Y

1841 ATAAAACCATTAATGACAAACCTATGATCTTGTTACCCGAAGGATGAATGGAAATTCTGCCTTATGATCAGCATTTCTTT 1920

K T I N D K P M I L L P E G * M E I L P Y D Q H F F

1921 TATGCTATACAAACTCACCAAGATCCTGGATCTTTAGATCTGGGATGAGAAAGTACTATTACTAAATTAAG-CAAAAATA 2000

Y A I Q T H Q D P G S L D L G * E S T I T K L ? Q K Y

2001 TTAGTGGCCTAGCCTTAGACTCACAGTTGATTCTGTCCTAGGATCCTGTGAGACCTGTTTGAATACACATTCAGCTAATG 2080

* W P S L R L T V D S V L G S C E T C L N T H S A N V

2081 TTTCATGACCACCTCCTTTCAAATTGGATAAACCTGAAAATCCATTTGATAAGATTTATATGGACTATGTTAGTCCCTTA 2160

S * P P P F K L D K P E N P F D K I Y M D Y V S P L

2161 CCTTCATCACAAGAGTATTTACATTTACTAGTTTTGGTAGACTATCTTACTGGATTTTTTTG-ATACACCCCACCAAAG- 2240

P S S Q E Y L H L L V L V D Y L T G F F ? I H P T K ?

2241 ATCAACTGCTAACACCATGGTTCATGCACTCTATGTATTCATCAGTGTAGCTATTCCAAAAGTAACCCATGCCGATCAAG 2320

S T A N T M V H A L Y V F I S V A I P K V T H A D Q G

2321 GTGCAGCTTTTACTTCAGGAACACTTACTAAGTGGGCTGCGGATCACAATATTGTATTGGAATTCAGCACGCCGTACCAT 2400

A A F T S G T L T K W A A D H N I V L E F S T P Y H

2401 CCCCAAAGTAAGGTGGAACAAAAAACTAGTGTCATAAAATGTGCACTAACCAAGCTGTTTGCAGGTCGACCTCAAAAGCG 2480

P Q S K V E Q K T S V I K C A L T K L F A G R P Q K R

2481 GTATCCTCTAATCCCTTTATTACAATTTGGAATTAATAATATACCTATTAATGTTGACAATGTAATCCCTTATCAGCTAT 2560

Y P L I P L L Q F G I N N I P I N V D N V I P Y Q L L

2561 TGTTTGGAGCTACACCAAACACCCCTTTTTCATAGGGTTCCAATTT--AACAACTAAGTTTAACAGCTGAACTTCGGCTA 2640

F G A T P N T P F S * G S N ? ? Q L S L T A E L R L

2641 A--CTTCTTCTTTCCACCCTGCCAGCCCCTCCAACACCTCCTAGCTCTTTCACCCTTCGGATTGACCTCTCTGTCCAGGA 2720

? L L L S T L P A P P T P P S S F T L R I D L S V Q E

2721 GATATACAGAAGGCACCTGC-TTTAAGGCCACATTGGAAAAAACCTACAAAGTTTCTTCAATTTTTAAATCCAAGGACTG 2800

I Y R R H L ? L R P H W K K P T K F L Q F L N P R T A

Env putative RF (uncertain starting site)

2801 CTATTGTTGAACATCTAGGCCAACCTAGAACTGTTAAGTATTGACAATTTAAG--GCCTACACCACATCAGCATCACACT 2880

I V E H L G Q P R T V K Y * Q F K ? P T P H Q H H T

I S I T L

End Pol putative RF

2881 ATCACTCATGGAATGGATGAGGTGGGATCTGGAAAGAAGACAGAGAGAGACTTATAAATATACTTCCTACGATCTTGAAA 2960

I T H G M D E V G S G K K T E R D L *

WXXW motif

S L M E W M R W D L E R R Q R E T Y K Y T S Y D L E S

2961 GCACACCCCCAGGGGTTTATGTACCAACAACCACCAAAATAACTGATGATGAACTTAAAGATCAGATCAGCTGGAAGGAC 3040

T P P G V Y V P T T T K I T D D E L K D Q I S W K D

3041 AAGTTTTGATATTACTGCTATGTGTCCTGTGCTAATACTACCAGGGTTATGACCAGCTATGTATCATT-TTAATACTCTT 3120

Hydrophobic transmembrane region

K F * Y Y C Y V S C A N T T R V M T S Y V S ? L I L L

3121 AGGTATCATTATTACTGCCAGCTTCATCACTATTTGTAGAATACAATGGCAACAAACCATAGAAGCTCAAGGCCCTACTA 3200

with a signal-peptide peptidase cleavage site

G I I I T A | S F I T I C R I Q W Q Q T I E A Q G P T I

3201 TATTTATAGGATCACATGAAGTTACTGTAAGAACACAGGGACAAATAATATCCAACTCTTCTTCTAATAGAAGGAAAATA 3280

Furin-cleavage site

F I G S H E V T V R T Q G Q I I S N S S S N R R K I

3281 GAAATCAAAGAAGACCCAAATATTACAGTCACCACTATACCACAAGGACATCATTGGGACCCATATCCAAAGCCTATTGA 3360

E I K E D P N I T V T T I P Q G H H W D P Y P K P I E

3361 GACCAAGGAGAGGGTCGTAGCTATATCACAAGTTCTGCTTCTAAATACAAAGAGTATATTAGACGCTGCTAAAATAACTG 3440

T K E R V V A I S Q V L L L N T K S I L D A A K I T D

3441 ATTGGAAGATGAAAACTAAGATTAAAGCAGCCATGCATAAAGAAATGTAAGATTTAGCTGACCAAACAT--ATATTACCT 3520

W K M K T K I K A A M H K E M * D L A D Q T ? I L P

3521 ATGGAGGACTCCTTCAACCAAGCCGACTATGAAGATAAACTGTGTTTTGACAACTTTGGAGACTGTTACGTAGTCGATTA 3600

M E D S F N Q A D Y E D K L C F D N F G D C Y V V D Y

3601 TCAAGAAGAGAGAACCTAGCCTATAAAAGAGATTATACAAGATCAATGTCCTTGACCCAAAGTATTAAACTTTAAGTATA 3680

Q E E R T * P I K E I I Q D Q C P * P K V L N F K Y K

3681 AACAAAACTTAGAGTTCAGGGCATGGCTATATTTTAATGATCCTAAGGGAGGAGGACCTAGACAAACTTTATTAACACAT 3760

Q N L E F R A W L Y F N D P K G G G P R Q T L L T H

3761 ATTACTGGGGACACTCCTAGAATACATGCCTTTGAAAAACCATTTGAAGAAAAATCCTATAAAGGAGCCATGTTTTGTAC 3840

I T G D T P R I H A F E K P F E E K S Y K G A M F C T

3841 AAAAAATAGATATAGGAGTTAGTACAACCATGATCAAGAATTTGAAGAGAATAAAAGGCTGCTGAAAAACCTCTTTAAGG 3920

K N R Y R S * Y N H D Q E F E E N K R L L K N L F K E

3921 AACTTATGAATGAACAAGGACTTTTGAACGCCACTGCCTTTCCAGAAGACTGGCATGAGTAGGGAAAAGGTAAAATATTC 4000

L M N E Q G L L N A T A F P E D W H E * G K G K I F

4001 AGACCATTTAAAATGGATTCCTTTTTCAAGGAACCCGAATTAGTTATGCACCTAAACAGAACCTACGATGCTTTTAACTC 4080

R P F K M D S F F K E P E L V M H L N R T Y D A F N S

4081 ATGGGAAAATGACAACCCAAATTGGAATGCATCTTCTTACACTAAATGCTTTCAGGAAGTTGCAAATAATGTGCACTCCT 4160

W E N D N P N W N A S S Y T K C F Q E V A N N V H S Y

4161 ATAATTGTGATTTTCAGCATGCAAAGATGTTTGATAAAGCCAATATTATTTATCTAGATAAAGA-AAATCAGACTTTACA 4240

N C D F Q H A K M F D K A N I I Y L D K ? K S D F T

4241 TTATTTCCTAAATATCTTGATGGAAGAATACAATACAATCTTGGGCAATTAGTTTTTACTAATATCATGAAAGGACCTTC 4320

L F P K Y L D G R I Q Y N L G Q L V F T N I M K G P S

4321 ATTTATA--AATACCACTGATTACTGATAAGAATTATAACATCTATTCATTATACGGAATATGTAAGCAGAAAGCTAAAA 4400

F I ? I P L I T D K N Y N I Y S L Y G I C K Q K A K T

4401 CTATTCCTTTTCATGATGTACTATCACAATTAAGATCTTATACAAAAATGTATGGTGTGATAGACATAATCAGGAATTTA 4480

I P F H D V L S Q L R S Y T K M Y G V I D I I R N L

4481 GGAGGCTTTAATGCAATGCCATTAAATAAATCAGTAAATCCTAACCCACAACAGTGGAATATCCATCAAATAAGGAAGAA 4560

G G F N A M P L N K S V N P N P Q Q W N I H Q I R K K

4561 GCTATCTGCTAATATATATACTCTCTTTGAAGCTACTCATCTAA--GAAAAATCAATGAATAAATTAATCCAATTATAAG 4640

L S A N I Y T L F E A T H L ? E K S M N K L I Q L * D

4641 ATTGGAATAACCAGCAACTGGAAAATGGGTTTCATTTATTACAGACACATGTTATTACTCTATTTGAATCAACCAGATAT 4720

W N N Q Q L E N G F H L L Q T H V I T L F E S T R Y

4721 GACCTTCAAATTCTTATGGCAATCCAACAACAGTTGGAAAAAATTAAAAATCAAGC-AAGAATGGGACATATGGATTGTG 4800

D L Q I L M A I Q Q Q L E K I K N Q ? K N G T Y G L C

4801 CGAATGGCATAGTGTC-ATGGAACTTGGCTCCAAGAACAACTAAATCTAACAGATGCACAAATGACTTTAGTTAAAGAAA 4880

E W H S V ? G T W L Q E Q L N L T D A Q M T L V K E T

4881 CATCTTCTGCAAGAATTAATAATGTCATTATGGGAGAAGATCATTATGAAATTCACATACATTGTGAACTCATAATACCT 4960

S S A R I N N V I M G E D H Y E I H I H C E L I I P

```
4961 AAAGAAATCTATGCTACAAACCAGAAACTACTTAACTATCTTGTCTCTCAAAACCAACACCTGCTGAAACTCACTATTGG 5040
     K  E  I  Y  A  T  N  Q  K  L  L  N  Y  L  V  S  Q  N  Q  H  L  L  K  L  T  I  G

5041 TTCTCAGTATCAATACTTAAATTATGAACCACGAACAGGTCAGCTGAAATATTTACAAGTACAAGGATGTACTGAAGATA 5120
      S  Q  Y  Q  Y  L  N  Y  E  P  R  T  G  Q  L  K  Y  L  Q  V  Q  G  C  T  E  D  K

5121 AATTCCTTATGTGTGACAAGATTAAAGAAGTTCCTCCATGTGGAATTAGTAATAATATCAGTAACTGTCCTGTTTACGTT 5200
       F  L  M  C  D  K  I  K  E  V  P  P  C  G  I  S  N  N  I  S  N  C  P  V  Y  V

5201 CAAACAATAAAAGACAAGCCTTATTTAGAGATCATTCCTCTACAGAATAGTTCCTACATTATTTTGATTG-AAGAAGTAA 5280
     Q  T  I  K  D  K  P  Y  L  E  I  I  P  L  Q  N  S  S  Y  I  I  L  I  ?  R  S  N

5281 CTGTTATATTCCTGCATACCAACCTATTGTTATCACAACTAAGTATCCAGTACACTGTCAAGAAAACGTCCTATCCCCAC 5360
      C  Y  I  P  A  Y  Q  P  I  V  I  T  T  K  Y  P  V  H  C  Q  E  N  V  L  S  P  P

5361 CACTTGAGACTACAAAATATGTCTCATATCTGGTTTTAGATTTACCACAAATACACTGGAGACTTCCTCATCTTCTTGGA 5440
       L  E  T  T  K  Y  V  S  Y  L  V  L  D  L  P  Q  I  H  W  R  L  P  H  L  L  G

5441 ACTTTAACTGAACTAAAAGGATATAAATTGAAGTTTTACAACTTGTATGACAATCTGCAAGATATTCTTGACCAGATCAG 5520
     T  L  T  E  L  K  G  Y  K  L  K  F  Y  N  L  Y  D  N  L  Q  D  I  L  D  Q  I  R

5521 AAGTACTCTTCTAAGATACAACGTCCACGAAAGAGATTTGCTTGCTTGGCTGACCCAGGTCTCTAAAGCCCTTGAAGATA 5600
      S  T  L  L  R  Y  N  V  H  E  R  D  L  L  A  W  L  T  Q  V  S  K  A  L  E  D  I

5601 TTGTCCCAGCAGCAGTAAACTTAATCAGTAAGGTTACTGCAGGTATTACTAACCTATTTGGAGGAGGATATTCAATGCTT 5680
       V  P  A  A  V  N  L  I  S  K  V  T  A  G  I  T  N  L  F  G  G  G  Y  S  M  L

                                                                   Bel1 putative RF
5681 TTTTATTTAATATATATCTTTTTAACAGGTTTTGCTATAATTGTCCTGATATTTATTATCAAAATAGTATCATGGCTACC 5760
     F  Y  L  I  Y  I  F  L  T  G  F  A  I  I  V  L  I  F  I  I  K  I  V  S  W  L  P
                                                                             M  A  T

     End Env putative RF
5761 CAAGAAAAAGTCCAGCAAGTCATAGTAGTGATGCCCTCCACATCACATAGAAAGACCAGAGGACTTATGTATAAATCTTT 5840
      K  K  K  S  S  K  S  *
     Q  E  K  V  Q  Q  V  I  V  V  M  P  S  T  S  H  R  K  T  R  G  L  M  Y  K  S  F

5841 TTCTCACTCAAGCCTG-TAGTACCGAACAAGCTAAAAAATTTAATAATACATTATAATATAACCTCTTTTTATGAAAATA 5920
      S  H  S  S  L  ?  V  P  N  K  L  K  N  L  I  I  H  Y  N  I  T  S  F  Y  E  N  N

5921 ATAATTTTAAGTTACTCTTAGTCTATAAATTGTGTGGAAAATAATTTGTAACTCATCATTTAAAAACTACTTGGGAATTA 6000
       N  F  K  L  L  L  V  Y  K  L  C  G  K  *  F  V  T  H  H  L  K  T  T  W  E  L

6001 GAATATAAACTGTGAAGCATTTACAATATGTATCCTGGTAGTATTAACCCATTGTCTTCTGCTATAAGACATTGCACCTT 6080
     E  Y  K  L  *  S  I  Y  N  M  Y  P  G  S  I  N  P  L  S  S  A  I  R  H  C  T  L

6081 AACCTCTCATAGTGATCCGAATGAGGAAGGGCAACCCTCTATGAGGAGAAAGCCAATTTGCCCTCTGCATAGAATTACTA 6160
      T  S  H  S  D  P  N  E  E  G  Q  P  S  M  R  R  K  P  I  C  P  L  H  R  I  T  K

6161 AGTGGAGAAGAAAAATAAGGATGGATAATAACAGATCACACATATGTCCAGGACAATCCACAGATGCCAACCTTCGAGAT 6240
```

W R R K I R M D N N R S H I C P G Q S T D A N L R D

6241 GCTGCCCCTCAACCTAATATCAGCCTTGAACATCCTGGAGAAAATAGCAACAACTGTTTCCCTAACATCGCCCTGGAAGA 6320

A A P Q P N I S L E H P G E N S N N C F P N I A L E E

End Bel1 putative RF

6321 ACTGTGCAGCGAATTCAATGTTCAAGTTAATTAGAAATCCTTTTGGAATATATGGTATATGTACTACTTGTGA

L C S E F N V Q V N * K S F W N I W Y M Y Y L *
