# Supplementary figures and images for: Discovery of prosimian and afrotherian foamy viruses and potential cross species transmissions amidst stable and ancient mammalian co-evolution
Source: Retrovirology. 2014 Aug 4;11:61. doi: 10.1186/1742-4690-11-61 (PMC4261875; doi:10.1186/1742-4690-11-61)

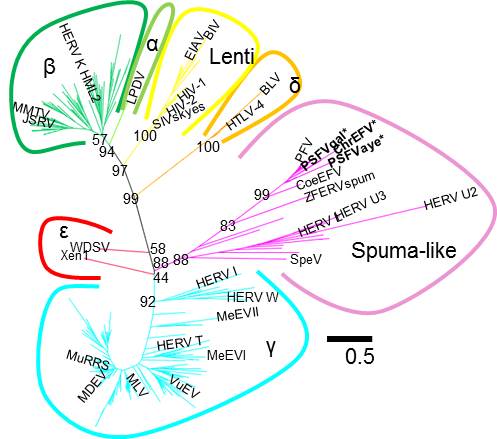

Supplement: Supplementary file 3 — Additional file 3: Figure S2: A consensus unrooted retrovirus reverse transcriptase phylogeny. The phylogeny was built using an alignment of reverse transcriptase proteins of several retrovirus genera: alpharetrovirus (α, light green), betaretrovirus (β, dark green), gammaretrovirus (γ, blue), deltaretrovirus (δ, orange), epsilonretrovirus (ϵ, red), lentiretrovirus (Lenti, yellow), and spuma-like retrovirus (Spuma-like, purple). The final alignment length consisted of 240 taxa and 162 amino acids in length. The tree was constructed using RAxML 7.2.8-HPC2 on XSEDE [83]. The best substitution model was determined by ProtTest 2.4 [84] to be the LG + G + F model. The scale bar is in units of amino acid substitution per site. Numbers on nodes are bootstrap support values estimated using 5,000 pseudoreplicates. PSFVgal, PSFVaye, and ChrEFV are indicated in bold type and with asterisks. JSRV, jaagsiekte sheep retrovirus; MMTV, mouse mammary tumor virus; HERV, human endogenous retrovirus (ERV); LPDV, lymphoproliferative disease virus; EIAV, equine infectious anemia virus; BIV, bovine immunodeficiency virus; HIV, human immunodeficiency virus; SIV, simian immunodeficiency virus; BLV, bovine leukemia virus; HTLV, human T-lymphotropic virus; PFV, prototype FV; CoeEFV, coelacanth EFV; ZFERV, zebrafish ERV; SpeV, sphenodon ERV; MeEV, Meles endogenous virus (EV); VuEV, Vulpes EV; MLV, murine leukemia virus; MDEV, Mus dunni EV; MuRRS, murine retrovirus-related sequence; Xen, Xenopus laevis ERV; WDSV, walleye dermal sarcoma virus. (JPG 34 KB) [file 12977_2014_3914_MOESM3_ESM.jpg]

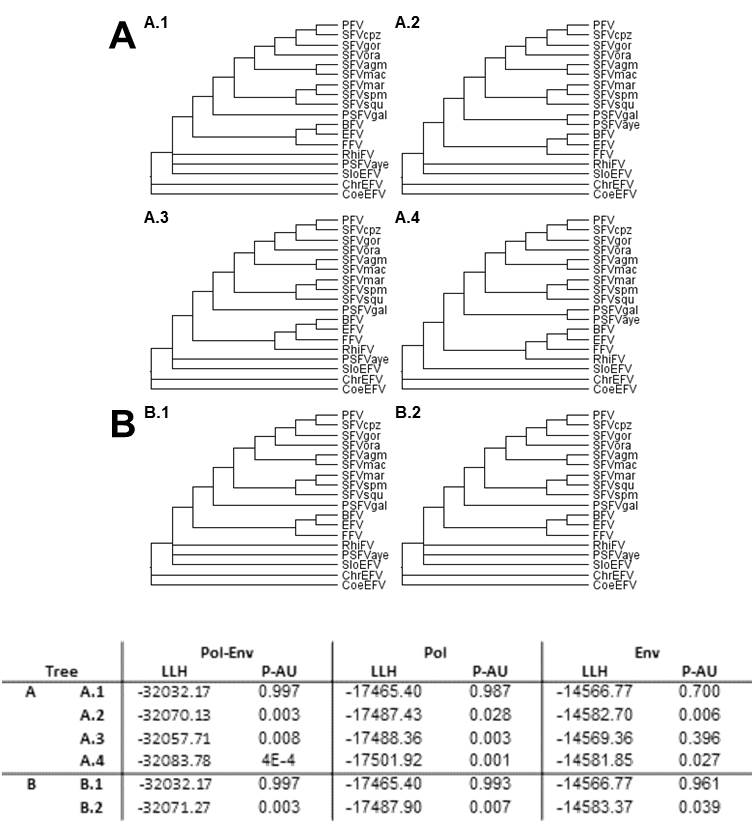

Supplement: Supplementary file 4 — Additional file 4: Figure S3: Foamy virus (FV)-host evolutionary conflicts. Our analyses suggested two major FV-host evolutionary conflicts: one is of PSFVaye and RhiFV (A), and the other is of New World monkey (NWM) FVs (B). A.1 shows our best estimated phylogeny in which both PSFVaye and RhiFV are placed robustly outside the Boreoeutherian FV clade but still remain together with exafroplacentalian FVs (see main text). A.2-4 show alternative phylogenies in which either PSFVaye or RhiFV (A.2 & A.3) or both (A.4) co-speciate with their hosts. We used approximately unbiased (AU) [67] tests to compare these alternative phylogenies against one another given Pol-Env/Pol/Env alignments, based on comparisons of site-wise log-likelihood scores (LLH) computed using PAML 4.7a [87]. The AU tests were performed in Consel [88]. The same analyses were performed to compare our best estimates of the phylogenetic placement of NWM FVs which show FV-host evolutionary conflicts (B.1) against topologies that do not (B.2). Results are summarized in the table; only total LLH scores and AU probabilities (p-AUs) are shown. (JPG 75 KB) [file 12977_2014_3914_MOESM4_ESM.jpg]

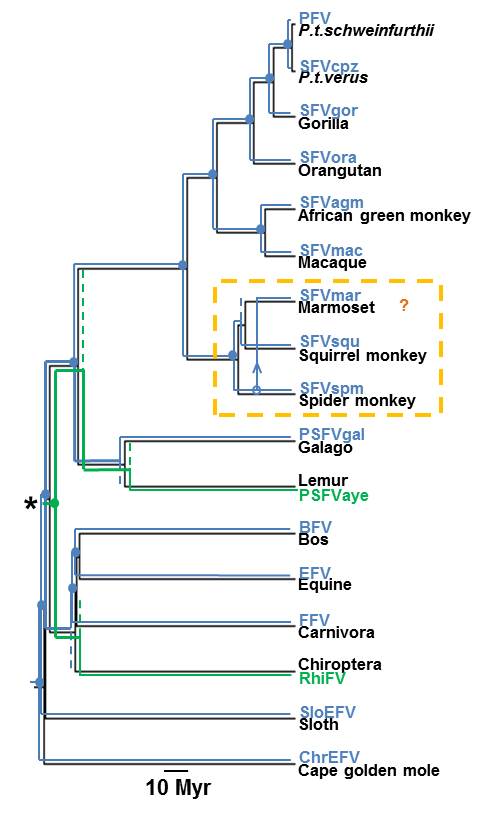

Supplement: Supplementary file 5 — Additional file 5: Figure S4: Inferred foamy virus (FV)-host co-speciation events using co-phylogeny reconstruction software Jane v4.0 [65]. The FV Pol-Env protein phylogeny (blue and green) was superimposed upon a previously published host phylogeny (black, published in [43]) and is scaled to host divergence times. The scale bar is in units of millions of years. Concatenated Pol-Env protein alignments (893 and 603 amino acids, respectively) were used to infer the FV trees. Solid circles represent FV-host co-speciation events, open circles represent host switching events, and an asterisk represents a lineage duplication event in the absence of host diversification which gave rise to two FV lineages sharing host species. The thick lines in the FV phylogeny correspond to the evolutionary period spanning about 30 Myr, during which the two lineages shared their host species. A hypothetical scenario of host switching of New World monkey FVs (as indicated by an orange ‘?’) is shown in an orange box. An alternative scenario is shown in Figure 4. (JPEG 43 kb) (JPG 44 KB) [file 12977_2014_3914_MOESM5_ESM.jpg]

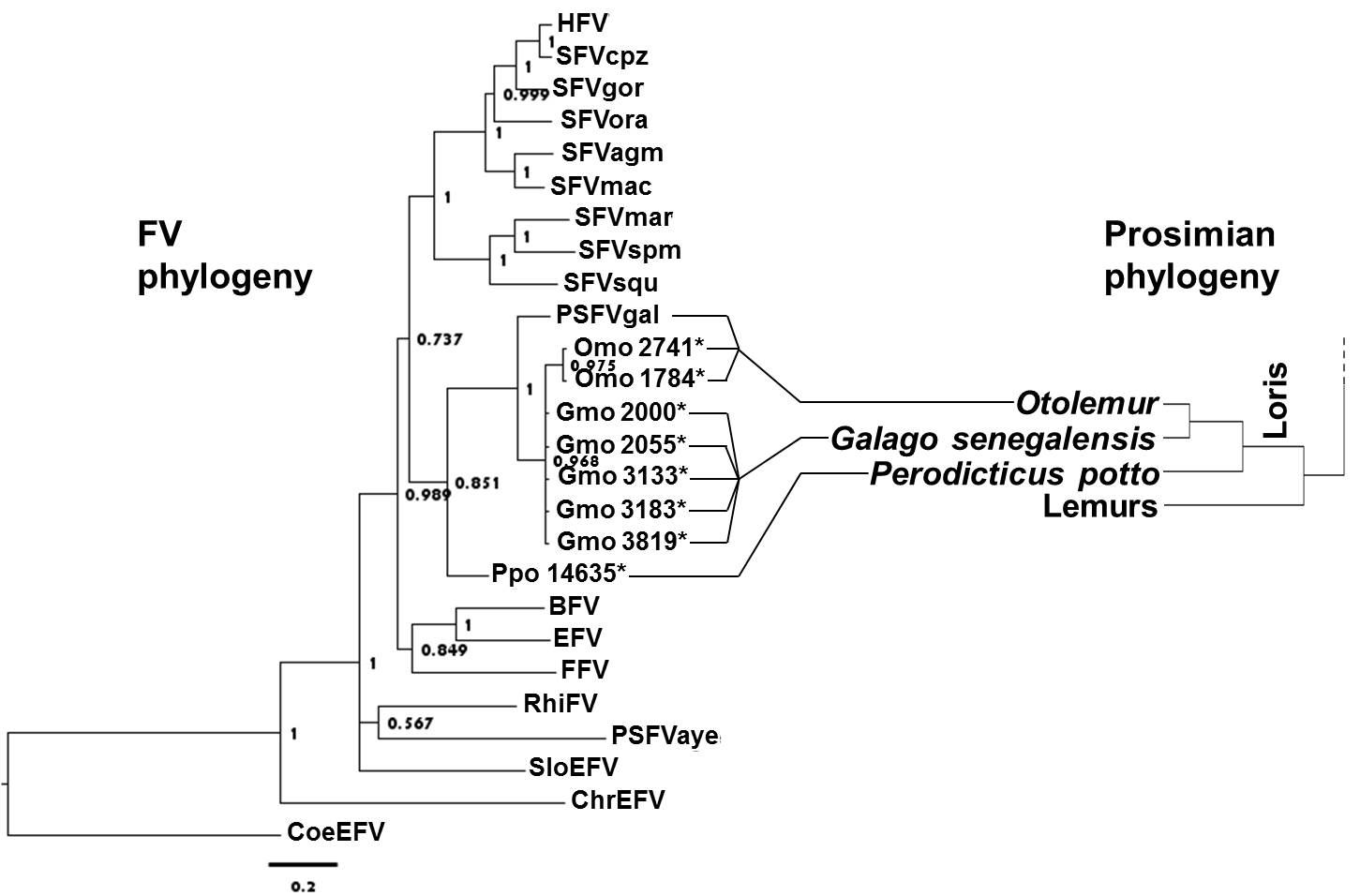

Supplement: Supplementary file 6 — Additional file 6: Figure S5: Co-speciation between lorisiforme foamy viruses (FVs) and their hosts. FV phylogeny (left) was estimated using an alignment of short integrase protein sequences of lorisiformes (indicated with asterisks, 85 amino acids (aa)) and concatenated polymerase-envelope protein sequences of other eutherian FVs (893 aa and 603 aa, respectively). The phylogeny was inferred using Bayesian methods in the program MrBayes 3.2.1 [85]) and rooted with CoeEFV. Numbers at branch nodes are posterior probabilities and the scale bar is in units of amino-acid substitutions per site. The topology of the lorisiforme FV phylogenetic relationships is compared to that of their prosimian hosts (right, previously published in [43]). Solid lines between the two trees indicate FV-host associations. (JPG 83 KB) [file 12977_2014_3914_MOESM6_ESM.jpg]

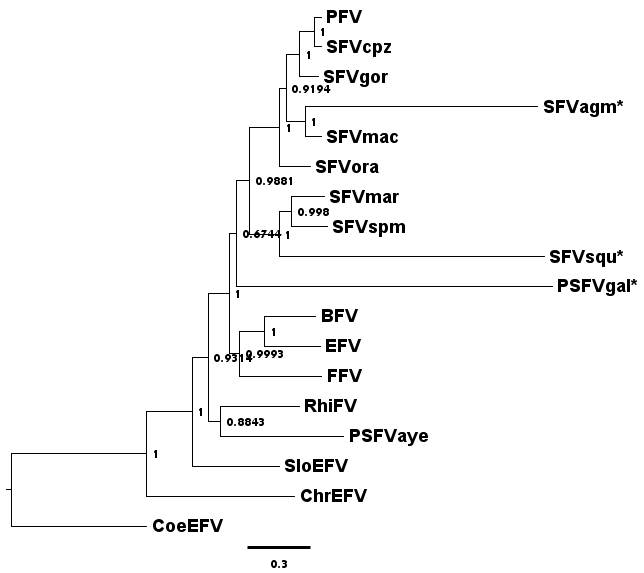

Supplement: Supplementary file 7 — Additional file 7: Figure S6: Investigation of the effects of neutral genetic changes on foamy virus (FV) phylogenetic relationships. We mutated the polymerase and envelope nucleotide sequences of SFVagm, SFVsqu, and PSFVgal (indicated with asterisks) in silico with a substitution rate of 10E-9 substitutions per site per year over a hypothetical period of 40 Myr. The mutated gene sequences were then translated into protein sequences and the FV phylogeny was re-built as described using Bayesian methods using MrBayes 3.2.1 [85]. The substitution process was assumed to be neutral, homogenous, and independent across all sites and base types. Numbers at branch nodes are posterior probabilities and the scale bar is in units of amino acid substitutions per site. (JPG 25 KB) [file 12977_2014_3914_MOESM7_ESM.jpg]
